# Prophylactic and therapeutic vaccine development: advancements and challenges

**DOI:** 10.1186/s43556-024-00222-x

**Published:** 2024-11-11

**Authors:** Induni Nayodhara Weerarathna, Elijah Skarlus Doelakeh, Lydia Kiwanuka, Praveen Kumar, Sanvi Arora

**Affiliations:** 1 Department of Biomedical Sciences, School of Allied Health Sciences, Datta Meghe Institute of Higher Education and Research (Deemed to Be University), Wardha, Maharashtra 442001 India; 2 Department of Anesthesia, School of Allied Health Sciences, Datta Meghe Institute of Higher Education and Research (Deemed to Be University), Wardha, Maharashtra 442001 India; 3 Department of Medical Radiology and Imaging Technology, School of Allied Health Sciences, Datta Meghe Institute of Higher Education and Research (Deemed to Be University), Wardha, Maharashtra 442001 India; 4 Department of Computer Science and Medical Engineering, FEAT, Datta Meghe Institute of Higher Education and Research (Deemed to Be University), Wardha, Maharashtra 442001 India; 5 Faculty of Medicine, Jawaharlal Medical College, Datta Meghe Institute of Higher Education and Research (Deemed to Be University), Wardha, Maharashtra 442001 India

**Keywords:** Prophylactic, Therapeutic, Vaccines, Vaccine development, Biomedical technologies, Advancements, Challenges, Ethical considerations

## Abstract

Biomedical research is fundamental in developing preventive and therapeutic vaccines, serving as a cornerstone of global public health. This review explores the key concepts, methodologies, tools, and challenges in the vaccine development landscape, focusing on transitioning from basic biomedical sciences to clinical applications. Foundational disciplines such as virology, immunology, and molecular biology lay the groundwork for vaccine creation, while recent innovations like messenger RNA (mRNA) technology and reverse vaccinology have transformed the field. Additionally, it highlights the role of pharmaceutical advancements in translating lab discoveries into clinical solutions. Techniques like CRISPR-Cas9, genome sequencing, monoclonal antibodies, and computational modeling have significantly enhanced vaccine precision and efficacy, expediting the development of vaccines against infectious diseases. The review also discusses challenges that continue to hinder progress, including stringent regulatory pathways, vaccine hesitancy, and the rapid emergence of new pathogens. These obstacles underscore the need for interdisciplinary collaboration and the adoption of innovative strategies. Integrating personalized medicine, nanotechnology, and artificial intelligence is expected to revolutionize vaccine science further. By embracing these advancements, biomedical research has the potential to overcome existing challenges and usher in a new era of therapeutic and prophylactic vaccines, ultimately improving global health outcomes. This review emphasizes the critical role of vaccines in combating current and future health threats, advocating for continued investment in biomedical science and technology.

## Introduction

Biomedical science encompasses fields such as biology, chemistry, physics, and engineering. It enhances our understanding of diseases, formulates remedies, and prevents pandemics [1]. A pandemic is an epidemic affecting large areas, multiple countries, or the entire world, causing more havoc than smaller, seasonal outbreaks. They tend to cause more havoc than smaller seasonal outbreaks or epidemics [2]. There is a strong association between using biomedical science to interpret the pandemics. Biomedical science is critical in interpreting pandemics by studying pathogens and their effects and developing diagnostic tools. For example, during the COVID-19 pandemic, biomedical scientists researched virus transmission, provided health guidelines, and developed surveillance techniques to curb the outbreak. This underscores the importance of integrating biomedical science into medical education. It also helps study the pandemic, the pathogen, and its effects [3]. One of the new budding applications in biomedical science to put a stop to the pandemic is diagnostic tool development. A recent case can be seen with the example of COVID-19 [4, 5]. Biomedical scientists have put their time into researching the transmission of the viruses. This gives valuable knowledge to develop health-related guidelines for the public to curb the virus's transmission and halt the outbreak. Researchers have also focused on looking into ways to detect the transmission of the virus. This involves various surveillance techniques that again help stop the outbreak before it gets widespread [6]. The rapid development of COVID-19 vaccines, particularly the mRNA-based vaccines, highlights the potential to apply similar strategies to other serious diseases. However, market uncertainty and lack of awareness can complicate the vaccine development. Understanding vaccination, introducing non-active disease-causing organisms, and the historical advancements in vaccine development are crucial for future progress [7].

The vaccine development process for COVID-19 showed the speed of development and has led us to focus on similar strategies for other serious diseases [8–10]. The path to vaccine development can be simple or highly complex. However, the factors that may complicate the entire process are market uncertainty or lack of awareness of the path for vaccine development [11]. As to what vaccination is, it is the provision of a non-active disease-causing organism to the living body. Vaccination, especially the one with the traditional development process, did not garner much attention in comparison with the vaccines developed by newer mRNA-based technology [10]. Vaccination helps in the induction of the antibodies and the T cell response. This helps keep the person safe against a variety of disease-causing organisms. These vaccines can be prophylactic or therapeutic. Prophylactic vaccines are preventive, while therapeutic vaccines are used post-infection [12].

Experience and observation to practice 'variolation' were used by people in the twelfth and fifteenth centuries [13], and the practice of variolation was the first known form of immunization of humans. Fluid or powdered scabs from the blisters of a smallpox patient were transferred into the recipient's skin via minor scratches on the surface. This technique was employed in diverse formats across China, the Middle East, and Africa before it was eventually transmitted to Europe in the seventeenth century. Edward Jenner inaugurated the seminal scientific investigation in 1796, using smallpox scabs and viruses from cowpox instead of mystery materials and performing human experiments based on Enlightenment principles. The vaccination process also got its name from Jenner; this marks the dawn of vaccinology [14]. Creating a vaccine usually involves a decade to fifteen years of research conducted predominantly within commercial entities' laboratories, often necessitating collaborations with university-based researchers.

The concept of vaccination came up in Europe in the eighteenth and nineteenth centuries, and significant advancements were made in the twentieth century due to developments in molecular biology and biotechnology [5]. Computational technology has also added to the development of vaccines. Programs and algorithms (Vaxign, Bowman-Heinson, VacSol, NERVE) are built around bacterial and prokaryotic systems that aid the process [15]. The ultimate aim is to target diseases like the Human Immunodeficiency Virus, Ebola, Zika virus, severe acute respiratory syndrome, dengue, chikungunya, and all cancer types: hepatitis and tuberculosis. However, on the downside, this has thrown light on the need for growth in the vaccine development market in the past 40 years [12, 16].

This review highlights the development of prophylactic and therapeutic vaccine processes with advancements and challenges. This article aims to provide a comprehensive overview of the latest advancements and challenges in developing prophylactic and therapeutic vaccines. It explores innovative vaccine technologies, such as mRNA and viral vectors, while addressing critical scientific, regulatory, and societal challenges. The article also highlights the ethical considerations and public perception issues that impact vaccine development and deployment, emphasizing the critical role of vaccines in modern medicine and public health.

## Fundamental concepts of prophylactic and therapeutic vaccine

Immunity is the body's ability to protect itself and withstand against damaging foreign bodies. It can be innate immunity or acquired immunity. The underlying principles of preventive and therapeutic vaccinations stem from the body's immune response, which can be classified into innate immunity and adaptive immunity. Innate immunity is the body's first shield against foreign constituents. It has a similar response to germs and foreign bodies. It is also called as "non-specific" immunity [17]. It is not because of a vaccine or an infection. This can be via physical barriers or internal defenses, namely skin, hair, cilia, mucus membranes, stomach acid, digestive enzymes in the mouth, and so on [18]. In the case of vaccinations, this initial reaction is crucial because it aids in stimulating the immune system as a whole, laying the groundwork for the longer-lasting and more focused adaptive immune response. For vaccines to effectively mount a defense against infections or diseases, they must activate the immune system during this early stage, both preventive and therapeutic [19].

The adaptive immune system is the system that provides a specific type of immunity and is based on memory. Typical features include specificity, heterogeneity, and memory [20]. The success of vaccinations is primarily dependent on the adaptive immune system, which provides long-term protection and is very specialized. The two primary components of a focused immune response that the body mounts in response to a pathogen or vaccine antigen are B cells, which generate antibodies, and T cells, which either directly attack diseased or infected cells or work together to coordinate the immune response. T cells are made in the bone marrow and use chemical messengers to activate other immune system cells. B cells are made in bone marrow but are activated by T cells. They turn into memory cells and help with the memory component of adaptive immunity [17].

Establishing immunological memory is a critical component of adaptive immunity, enabling the immune system to react to disease more quickly and efficiently the next time. This idea is essential to the goal of preventive vaccinations, designed to create memory B and T cells that remain in the body to create long-lasting protection. This is a function of specificity and longevity [21]. The Greeks first recorded this, and it has now become a vaccine. The memory is maintained by long-lived antigen-specific lymphocytes brought in by the first exposure and continues until a second interaction with the pathogen. This memory can be measured as adoptive transfer assays of lymphocytes. The entire process comprises an initial response by a primary antibody and then a secondary antibody, which comes in later. The production of IgM, IgG, IgA, and IgE marks the secondary antibody response. A secondary immune response will come into play upon a second interaction with the same antigen [22].

These memory cells can quickly create a defense to stop the sickness from spreading when they come into contact with the same infection again. Though they frequently aim to elicit a more rapid and robust immune response, mainly when the disease is already established, like in chronic infections or cancer, therapeutic vaccines also use immunological memory. Depending on the vaccination's intended use, the ultimate objective in each scenario is to teach the immune system how to react to particular antigens more successfully, either for preventive or therapeutic purposes. A thorough understanding of the molecular mechanisms behind immune activation and the biological mechanisms of infections is crucial for creating preventive and therapeutic vaccines. The development of vaccines depends on figuring out the distinct features of pathogens, like viruses or bacteria, that can be attacked to trigger a potent and successful immune response. Scholars in virology, pathogen biology, and molecular biology can identify particular antigens that elicit the immune system to identify, combat, and retain the infection. This knowledge helps develop therapeutic vaccinations that strengthen the immune system's capacity to combat pre-existing illnesses, including cancer and chronic infections, and vaccines that prevent infection [17].

Virology refers to the study of viruses and infections caused by them. They can infect all life forms (bacteria, plants, protozoa, fungi, insects, fish, reptiles, birds, and mammals). They are small, subcellular agents that cannot multiply out of a host cell. Thus, they are intracellular and obligate parasites. They can either have a viral Deoxyribonucleic Acid (DNA) or Ribonucleic Acid (RNA) and cause a variety of diseases like HIV (Human Immunodeficiency Virus), ebola, herpes, chicken pox and so on [23]. Pathogens, unlike general thinking, are not limited to hostility. It lives at the expense of the host [24]. In scientific terms, it is defined as an organism causing disease to its host, with the severity of the disease symptoms referred to as virulence [25]. They can be facultative or obligate. Obligate pathogens need a host to complete their life cycle; this can be one host or multiple. Facultative pathogens are mainly environmental bacteria or fungi, requiring a host only as a niche. Furthermore, knowing a pathogen's virulence, or the degree of the disease it causes, aids in developing vaccinations that either weaken the pathogen or expose the immune system to a safe form of its antigen [26].

Molecular biology is an essential field in vaccine development that studies the interactions between proteins, RNA, and DNA [27]. Nucleic acids are simpler than proteins since they comprise only four main types of bases joined via a chain of sugars and phosphates. The sequence of these bases in the DNA of any organism forms its genetic information. This sequence makes all of the proteins an organism can produce, all of the chemical reactions it can carry out, and all of the behavior the organism can showcase as an answer to its environment [28]. Researchers have also quickly found new antigens and enhanced vaccine formulation because of developments in molecular biology, such as genome sequencing [29].

Vaccines, whether preventive or curative, utilize the body's natural defenses against infections and illnesses, both innate and adaptive. The body's first line of defense is the innate immune response, while memory cells produced by the adaptive immune system give the body specialized, long-lasting protection [30]. Whether creating vaccines to prevent infections or cure pre-existing diseases like cancer and chronic infections, a thorough grasp of virology, pathogen biology, and molecular pathways is crucial. By making it possible to identify antigens and improve vaccine composition precisely, recent developments in molecular biology have sped up the research on vaccinations and held out hope for the day when vaccines may be customized for both therapeutic and preventive uses [31].

## Vaccine development process

Vaccines serve an essential role in world health. They contribute to longer, healthier lives and higher quality of life. Vaccination is an effective means to avoid several dangerous and contagious diseases. It has been identified as one of the most essential approaches to combat a pandemic [32]. Its applications include smallpox and polio eradication [33]. Vaccination has substantially decreased the prevalence of many pediatric illnesses, including measles and polio [34]. Influenza vaccination is routinely used yearly to guarantee influenza prevention during the season [35]. As a result, many scientific studies have recognized vaccination as one of the most efficacious disease transmission prevention measures through stopping disease transmission. Infectious diseases as human health problems are one of the biggest concerns in healthcare [36]. Terrifying are the diseases that spread around, especially those that kill many victims. Traditionally, viral infections were considered among the misfortunes or the expressions of a deity's wrath from the earliest times of humanity [37].

Advancements in the etiological study of diseases caused by viruses, knowledge of microbiology, and the discovery of different vaccines have prevented humanity from its unreasonable fear of death. The invention of vaccines and their development is regarded as one of the most outstanding achievements in medical history. Millions of lives have been saved because of vaccination, and its value is only expanding. Despite extensive efforts that have been made to develop competent and productive vaccines, there are inadequate obstacles to protecting people from diseases that could produce outbreaks or widespread diseases affecting a large number of people [38, 39]. Therefore, researchers are attempting to extend the number of illnesses that vaccinations can stop, thereby broadening the target populations who will benefit from vaccination in the future. Moreover, the techniques of developing vaccines are customized to meet individual countries' distinct wellness and financial needs. Before introducing a vaccine to the community, some initial stages should be gone through, such as proof of concept, vaccine testing, and the final vaccine manufacturing process.

### Proof of concept of vaccine development

As with other forms of prevention, health organizations must conduct relentless, post-licensure safety testing by active and passive methods of the vaccines distributed worldwide. Academia should investigate vaccine safety and concentrate chiefly on manufacturing vaccines and letting vaccine manufacturing be the production of the vaccines. Above all, how people see vaccine-making can affect their faith in and usage of approved vaccines [40, 41]. However, this priority should not be confused with the notion that strict quality control practices must also characterize the vaccine production process. The general public's perspective on vaccine manufacturing can either make people take up the vaccination or lose faith in these vaccines entirely [42, 43]. For example, improved knowledge of the conditions and potential complications in the case of lack of vaccine, such as the flu or human papillomavirus (HPV), and the mechanism of vaccine are likely to shape public opinion and stimulate public responsiveness to vaccination programs [44, 45].

The manufacturing of vaccines, as with any other medical product, must pass good manufacturing practices, but it has several quality control stages more than any other pharmaceutical [46, 47]. Vaccines are produced within 6–24 months. At the same time, the whole process, including all of the developmental stages, usually lasts many years, mainly because its quality control and testing procedures are very demanding [48]. The company must ensure that it has established and practiced quality tests, including the purity of the cell substrate, in every production phase. Assessments such as testing the quality of the organism harvest, ensuring the whole manufacturing process is practical, and making sure the suitability when it is taken into large containers are included [49]. The pharmaceutical industry must follow this strict guideline, placing the primary legal responsibility on the manufacturer to ensure their product's safety, quality, and efficacy [50]. If the vaccine demonstrates promising results, it will advance to clinical trials involving human subjects.

### Vaccine testing

The vaccine progresses to the clinical testing stage, often called a clinical trial. The process starts early here when the company presents an IND (Investigational New Drug) application to the FDA (Food and Drug Administration); this application contains the data gathered from animal studies, the manufacturing process, and research details about the vaccine quality. The quality assurance of vaccines plays a role of primary importance as it heavily influences the vaccine's effectiveness during both short- and long-term periods of disease prevention. The clinical trial process is a three-stage puzzle set, and an additional fourth phase of the FDA will be considered if the agency approves the vaccine [51]. These days, biological systems are handled and analyzed using newly developed technologies that are revolutionizing information technology and biotechnology and generating enormous volumes of data.

Integrating this data is necessary to expedite the process of knowledge discovery and facilitate the study of biological systems at different levels. *Modeling* is how humans represent, manipulate, and communicate everyday items seen in the real world. Increasingly, high-throughput genomics and proteomics efforts generate biomedical data that can be deduced using mathematical and computer models. Using sophisticated computer models that simulate intricate biological processes results in the formulation of theories and the suggestion of investigations. Computational models are prepared to use text mining and knowledge discovery techniques to exploit the abundance of data on biomedical databases.

The 1980s saw the development of the first immunoinformatics tools for vaccine creation by DeLisi, Berzofsky, and others. Epitope-mapping algorithms are the most critical informatics tool for vaccine design [52]. Accompanying the progress in molecular biology and sequencing technologies, in silico identification of vaccination targets has been made possible by bioinformatics analysis of microbial genome data. Hundreds of new vaccine design algorithms have been developed due to further developments in immunoinformatics. This new method of vaccine development is known as immunome-derived vaccine design or reverse vaccinology [53, 54].

Several computational modeling techniques, such as higher-order systems, agent-based models, and mathematical models, are applied to vaccinology science. Understanding biological systems requires the use of computational models. These models can forecast or improve therapy outcomes at the organism level. Agent-based modeling (ABM) can be used to conduct in silico experiments that result in the formulation and validation of biological hypotheses and provide helpful guidance for designing the best possible treatment plans.

SimTriplex is a specialized cellular automata successfully used in computational vaccinology to model the Triplex vaccine, mammary cancer, and immune system competition [55]. Triplex is an immune-preventive HER-2/neu breast cancer vaccine comprising two non-antigen specific adjuvants (IL-12 and allogeneic major histocompatibility complex (MHC) class I molecules) in addition to the specific target antigen, p185 (HER-2/neu). HER-2/neu transgenic mice were used to evaluate four different vaccine administration schedules: early, late, very late, and chronic. The results indicated that the chronic schedule only offers complete, long-term protection against mammary cancer [56]. Before manufacturing the vaccine, there are three clinical phases: phase one, phase two, and phase three.

#### Phase 1

In this trial stage, small samples of the participatory group serve as subjects (a variety of 20 to 100), and the first group will be vaccinated. This is the research phase where the investigators will examine the vaccine’s possible complications with the use of human subjects and find out if it can prompt immune responses [57].

#### Phase 2

The trial is extended from a single-digit triad to a three-digit care group with the selection of the samples that will be vaccinated once the trial is successful. This is based on other factors like age and physical condition. It also aims to include people from different backgrounds to ensure that all communities are represented. This testing stage aims to get further safety information related to potential side effects and dangers and to evaluate the immunogenicity of a vaccine [58].

#### Phase 3

The clinical trial is expanded to include a group of thousands of patients, ranging between 1,000 and 3,000. In this stage, scientists verify the efficiency of the vaccine, evaluate its common and rare side effects, and collect data that supports it as an approved human application. This is referred to as Phase Four after FDA approval has been granted. As soon as the FDA approves (also known as "licensing") the use of vaccines for the general population, it may move on to another phase of clinical trials with many participants involved. Phase four is designed as a structured continuous study that assesses the safety and effectiveness of new vaccines over long periods [59].

### The vaccine manufacturing process

**Fig. 1:**
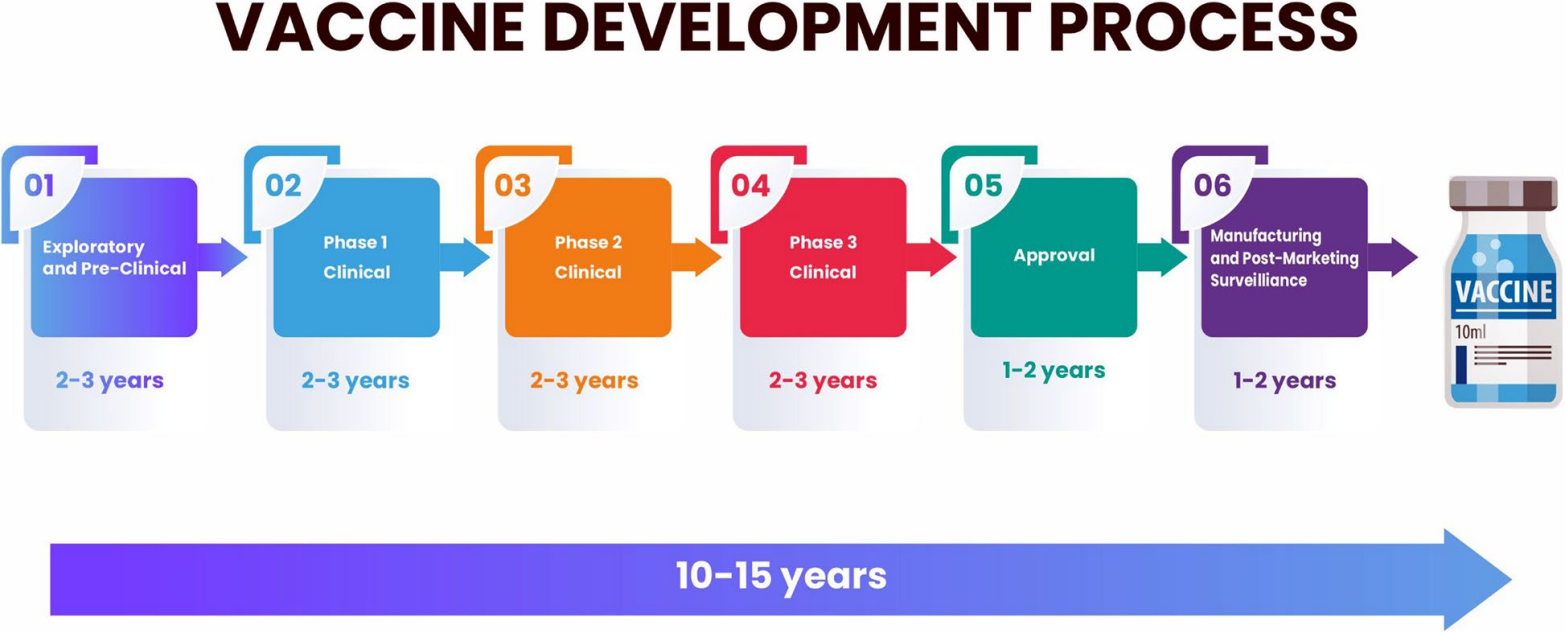
Schematic represents the vaccine development process

The FDA tries to see if the process of manufacturing the vaccine developer’s choice is up to standard in the last Phase 3 clinical trial. Besides this, the FDA audits/inspects the vaccine manufacturing facility to ensure a consistently dependable production of large quantities. The manufacturer produces lots, which, in turn, the approval authorities purposefully compare between each lot to verify uniformity. The FDA requires a firm to submit the data following the pre-clearance to exercise control over implementing a proven and efficacious production sequence [60]. Developing a vaccination can take ten to fifteen years, from beginning to end **(**Fig. 1**)**.

## Therapeutic vaccine development process

Immunotherapy through therapeutic vaccines is a viable and efficient approach for tackling new illnesses, chronic diseases, and existing epidemic and endemic diseases. They have safety, precision, in and out, and tumor enlargement can respond, so it is most effective. However, most therapeutic vaccines are still being tested in clinical trials to ensure they offer effective results. Nevertheless, better comprehension of the flow of immune reactions associated with the vaccines and burning immunomodulatory approaches could enhance the clinical applicability and efficacy of the vaccines [61]. Efforts to progress therapeutic vaccines should focus on three key areas: choosing an antigen, testing for an antigen, and deciding on the delivery method of antigens therapeutically or otherwise, which come in handy. It is also important to point out that searching for stable antigens associated with differing pathophysiologic events often requires a better understanding of pathophysiologic processes. For example, a better understanding of tumor-associated antigens, neoantigens, and natural immune responses has improved vaccine design in tumor immunology [62]. Regarding optimal results, the quality of neoepitopes should be improved more.

Antigen delivery is heavily influenced by the adjuvant used and the route of administration. Each form of therapeutic vaccination has distinct advantages and disadvantages. Protein/peptide vaccines are easy to produce but have limited immunogenicity. Vector-based vaccinations often deliver antigens with excellent effectiveness but raise safety issues. DNA vaccines lack a robust immunological response [63]. Activating adjuvants can boost the innate immune system's cellular and molecular components, thereby augmenting the efficacy of protein/peptide and DNA vaccines.

Moreover, emerging carrier systems such as lipoplex, liposomes, and self-assembling nanoparticles are anticipated to significantly contribute to developing protein/peptide vaccine platforms. Further, installing other/novel therapies like immune checkpoint inhibitors, CAR-T cell therapy, and TCR-T therapy shall also improve therapeutic vaccines. It has been found that therapeutic interfaces are enhanced when neoantigen vaccines are administered in combination with other immunotherapies. This is because, in chronic viral infections, the virus-specific T cells are gagged, letting them be exhausted quickly [64]. In vivo, inhibition of the PD-1/PD-L1 pathway positively impacted the efficacy of the therapeutic HBV (Hepatitis B virus) vaccine in the LCMV mouse model [65].

Chronic non-communicable diseases are a leading contributor to illness and death on a global scale, responsible for 71% of all fatalities and exerting a considerable impact on the global economic burden [66]. Chronic non-communicable diseases encompass conditions such as cardiovascular disease, cancer, respiratory illnesses, diabetes, hypertension, Alzheimer's disease, dyslipidemia, asthma, and chronic obstructive pulmonary disease [67]. Over 80% of fatalities stem from the leading quartet of chronic non-communicable illnesses, comprising cardiovascular diseases, cancer, respiratory ailments, and diabetes [6]. Thus, therapeutic vaccine development is desirable for an established disease that can go around the body's immune system to achieve a targeted immune response and constant destruction of the known disease. These vaccines provoke antigen-specific immune responses to prevent infected cells from escaping elimination [68]. In 1890, Koch developed the therapeutic vaccine for Tuberculosis (TB) known as Tuberculin, which consists of Tuberculin suspended in glycerol to create a preparation. This marked a significant advancement in distinguishing vaccines' preventive and healing capabilities [69]. Following Almroth Wright's depiction of employing a comparable antibacterial immunization for addressing prolonged regional bacterial infections in 1897, therapeutic vaccines underwent swift advancement yet simultaneously provoked considerable controversy due to misuse [70].

**Fig. 2:**
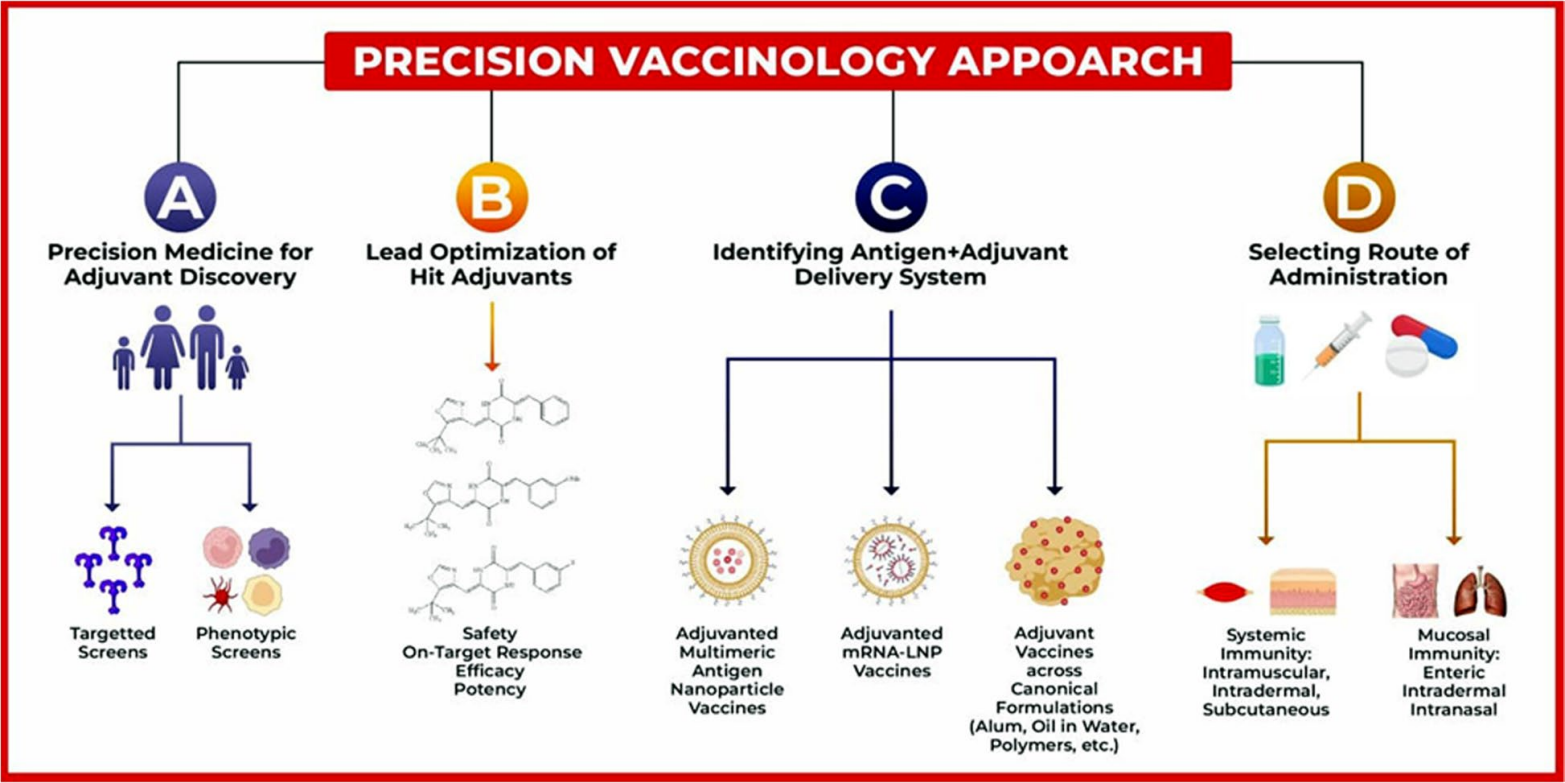
The precision-vaccinology strategy for responding to and preparing for epidemics or pandemics involves: Includes: **A** particular selection of strong adjuvants fitting the target population, **B** modification of the chemical structure of certain adjuvants for specific functions, **C** optimization of the use of mixtures of antigens and adjuvants combined with appropriate formulation, **D** identification of the right pathway of administration capable of adequately targeting protective correlates

The different compounds and antibiotics have affected the researchers' challenge of therapeutic vaccines. Since HIV was first discovered in early 1981, antiviral immunology has been rapidly advancing. Healthcare reforms, changes in disease nature and patterns, particularly of chronic diseases, and the growing prevalence of antibiotic-resistant bacteria have influenced therapeutic vaccine development again intensively after the approval of Sipuleucel-T in 2010 [71], The development of therapeutic vaccines gained momentum, with numerous studies demonstrating their beneficial clinical role in treating tumors and infectious diseases [72]. Based on fundamental research, they have begun moving towards the stage of clinical trials, and this reflects the scholarly literature foundations. Currently, many therapeutic vaccines are under clinical trials for therapeutic uses in both communicable and non-communicable illnesses, infective and non-infective, such as viral diseases, cancer, hypertension, Alzheimer's disease, diabetes, and dyslipidemia [73]. Therapeutic vaccinations offer numerous advantages compared to contemporary chemical or biological treatments. These include precise targeting, minimal adverse effects, enduring efficacy, and immunity to drug resistance. They present a promising avenue for combating infectious diseases and chronic non-communicable conditions, offering renewed optimism for treatment [74]. Figure 2 shows the precision vaccinology approach.

### Clinical trials

It is crucial to have clinical trials in the development of vaccines. They provide safety and efficacy for regulatory purposes and health management, supporting the NITAG (National Immunization Technical Advisory Group). The design of the vaccine clinical program and specific trials that make up this program is a process that can only be conceptualized and mapped out in advance in many cases. This includes preclinical data, epidemiological information, other vaccines and medical treatments intended for the targeted product profile, and new clinical data as they become available. Further characteristics of vaccine clinical trials involve assessing tolerance and side effects to assess safety; utilizing serologic or immunologic changes as markers of biological effect; targeting mainly to avoid naturally occurring infective illnesses as the significant test of effectiveness; incorporating indicative effectiveness pointers short of prevention of the disease; and utilizing clinical records to ensure drug quality and consistency [75].

### Therapeutic vaccine

Therapeutic vaccines are categorized into three types, suitable for preclinical and clinical trials: molecular-based vaccines, vaccines delivered with the help of vectors, and vaccines created with the help of living cells [76]. Molecular-based immunizations include peptide/ protein vaccines, which contain entire proteins or protein portions of an illness-causing agent; DNA vaccines, which use the tiny genetic material of the pathogen; and mRNA vaccines [77]. These notable immunizations employ neoantigens, pure peptides/proteins, or genes encoding the proteins-DNA/mRNA in combination with adjuvants to generate immune responses [78]. Vector-based vaccines utilize naturally existing or genetically engineered bacteria, viruses, and yeast as effective transporters of antigen transgenes [79, 80]. Dendritic cell vaccines and genetically modified cell vaccines are two types of cell-based vaccinations that express or transport antigens using dendritic cells or cells that have been genetically modified [81, 82]. Immunomodulating vaccines administer antigens intravenously or extraneously with adjuvants to prime dendritic cells (DCs). These vaccinations effectively target existing antigens by improving the appearance of epitope-specific T cells to get to the site of infection and lesions to halt the infection. Also, they activate specific B cells in the body to secrete antibodies specific to the virus, which it has to neutralize [83]. Table 1 describes the types of therapeutic vaccines and their sources and applications.

**Table 1:** Overview of current therapeutic vaccines: sources, applications

**Serial No**	**Vaccines**	**Sources**	**Application**	**References**
1	Provenge (sipuleucel-T)	It is a therapeutic cancer vaccine developed by Dendreon Corporation	This treatment is uniquely crafted to combat prostate cancer by activating the patient's immune system to identify and eliminate prostate cancer cells	[84]
2	Talimogene laherparepvec (T-VEC)	It is is an oncolytic viral therapy derived from the herpes simplex virus type 1 (HSV-1)	This genetically engineered organism is designed to replicate specifically within tumors and generate the immune-boosting protein granulocyte–macrophage colony-stimulating factor (GM-CSF), aiding in the eradication of cancerous cells	[85]
3	Cervarix	It is a vaccine developed by GlaxoSmithKline (GSK)	is a vaccine developed by GSK. It is primarily used to protect against HPV types 16 and 18, which are associated with a significant risk of cervical cancer and has been research into its therapeutic potential in treating existing HPV-related conditions	[86]
4	BCG vaccine (Bacillus Calmette-Guerin)	It is derived from a strain of Mycobacterium bovis, which is a relative of Mycobacterium tuberculosis, the bacterium that causes tuberculosis	The BCG vaccine is primarily used to prevent TB and is also employed in the treatment of bladder cancer	[87]
5	VGX-3100	is a therapeutic DNA vaccine developed by Inovio Pharmaceuticals	designed to treat precancerous lesions caused by HPV types 16 and 18. It targets the E6 and E7 oncoproteins of HPV, which are crucial for the virus's role in causing cervical intraepithelial neoplasia (CIN) 2/3 lesions	[88]
6	HIV therapeutic vaccines	ACTG's A5374 Study AELIX-002 Study NIAID Research	Help to manage the virus without the need for continuous antiretroviral therapy (ART)	[89]
7	Hepatitis B therapeutic vaccines	INOVIO Pharmaceuticals Dynavax Technologies	designed to treat individuals already infected with the HBV, aiming to enhance the body's immune response to control or eliminate the virus	[90]
8	CIMAvax-EGF	It is a therapeutic cancer vaccine developed by the Center of Molecular Immunology (CIM) in Havana, Cuba	It is designed to treat non-small cell lung cancer by targeting the epidermal growth factor (EGF), which is involved in the growth and proliferation of cancer cells	[91]

### Immune response induced by therapeutic vaccines

Strategically administered vaccines such as bacterial and viral vectors, peptide/protein, and DNA and mRNA vaccines are delivered locally. Adjuvants in these vaccines activate local innate immune cells and appear to induce specific inflammatory mediators' chemokines [C/C] and [X/X] that attract macrophages and DCs to the injection site. The dendritic cells in the skin capture the subcutaneous antigen in the vaccine, and after being activated, the cells move to the regional lymph nodes. DC maturation results in the expression of MHCII molecules and co-stimulatory receptors – CD40, CD80, and CD86 – on their surface and the release of cytokines. In nodes of lymphatic tissue, the activated DCs then display the specificity antigens, which are presented in the form of peptide-MHC to T lymphocytes. At the same time, co-stimulatory and cytokines signals activate the CD8 + T cells. As the center of focus, the Ag-stimulated mature B cells differentiated antibody-producing cells and CD8 + T cells specific to the particular Ag disseminate to the focus of infection/lesion and perform their functions [81]. Adjuvants are essential in vaccines because they help increase the intensity and sustainability of the immune response to antigens. The commonplace adjuvants of approved vaccines include alum, MF59, CpG 1018, AS01, AS02, AS03, and AS04; other effective adjuvants are still under study in animal models and human tests. These substances, which bear immune stimulating characteristics, can either be like the damage-associated host-derived molecules or pathogen-derived molecular patterns that engage with Pattern Recognition Receptors (PRRs) on innate immune cells [92]. In clinical settings, therapeutic vaccinations are commonly given via intramuscular, subcutaneous, or intradermal routes, with intramuscular injection being the most commonly used method [93]. Influence on the potency and duration of the adaptive immune response is exerted by inflammation at the localized tissue [94].

**Fig. 3:**
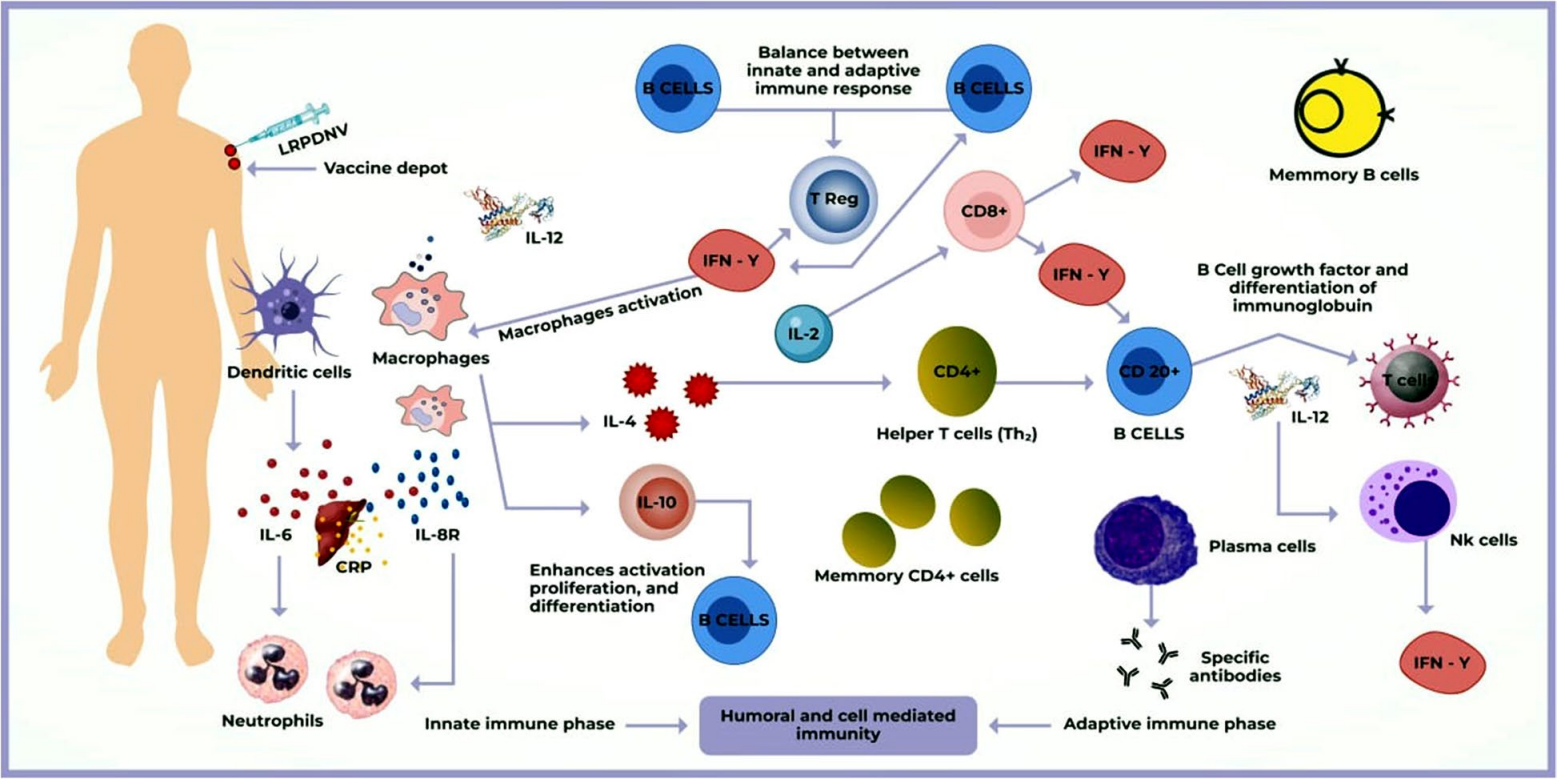
Schematic representation of vaccine efficacy and safety are governed by innate and adaptive immune responses. Host variables influence these interactions and can be guided by vaccination characteristics

Adjuvants in the vaccines elicit an immune response that causes the activation of the local innate immune responses and release chemokines, including CCL2 and CXCL1, among others, and cytokines. These substances then recruit more innate immune cells, including neutrophils, macrophages, monocytes, and dendritic cells, to the injection site [27, 28]. DCs enter the injection site and act as the primary antigen-presenting cells (APCs), triggering a robust adaptive immune response [93]. DCs at the immunization site endocytose the vaccine's antigen component. DCs stimulated at the immunization site transport vaccine components to the draining lymph nodes [95]. Appropriate stimulation of the dendritic cells needs to increase the surface expression in MHCII and CD40, CD80, and CD86 molecules and cytokine synthesis. Mobiles and activated dendritic cells bear antigens in the form of peptide MHC complexes to the T lymphocytes within the lymph nodes. Classically, CD8 + T cells are known to be activated only when they receive co-stimulatory and cytokine signals [96]. B cells that antigens have activated undergo maturation and rapid proliferation. Subsequently, CD8 + T lymphocytes, specific to the antigen, migrate to sites of infection and lesions to carry out their function**.** Figure 3 depicts the complex network of cells and mechanisms that make up the body's immune system.

## Vaccine development technologies and innovations

**Fig. 4:**
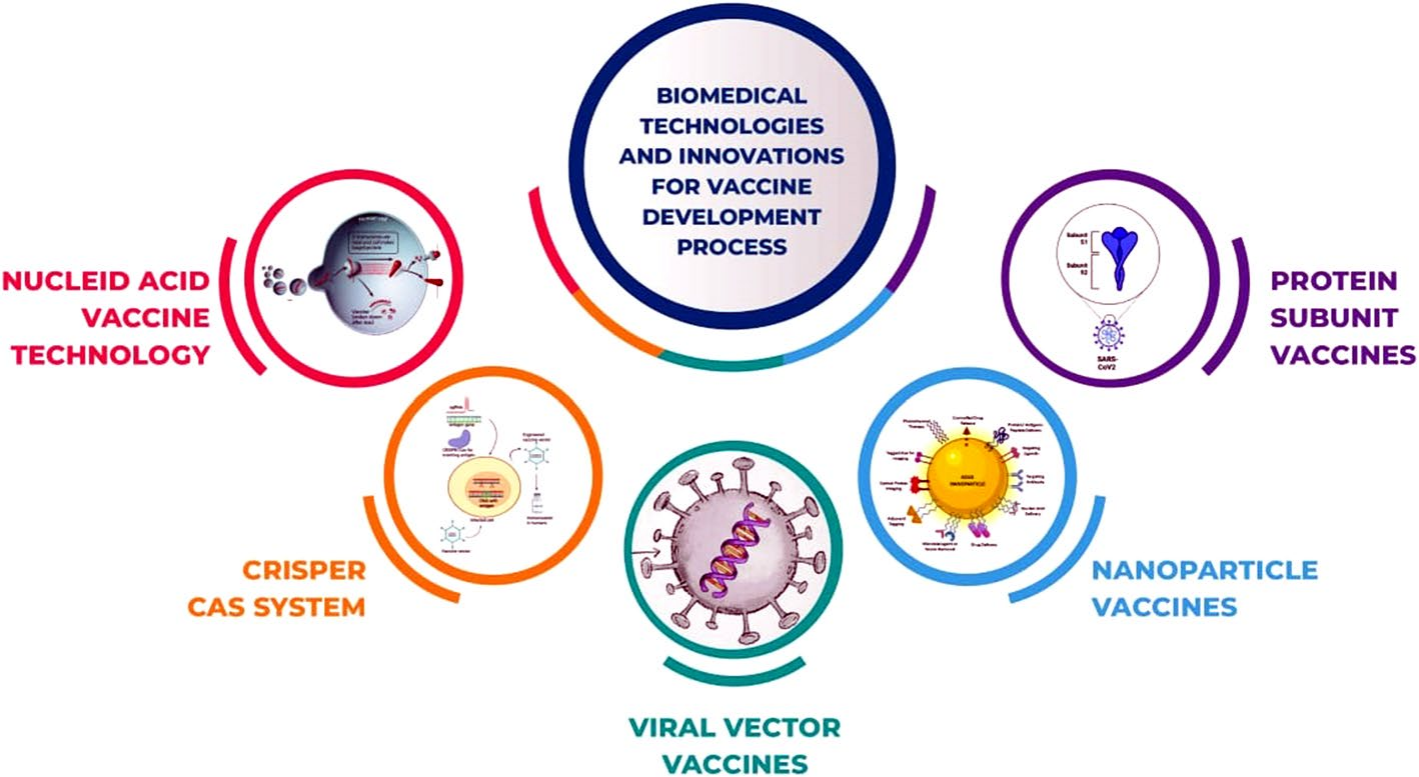
Various development technologies and innovations in the vaccine development process, including nucleic acid vaccine technology, protein subunit vaccines, nanoparticle vaccines, viral vector vaccines, and the CRISPR-Cas system, highlighting the diverse approaches used to enhance vaccine efficacy and development

Many advanced technologies and advances have revolutionized the vaccine development landscape. These developments cover various approaches, such as CRISPR-cas systems, protein subunit vaccines, nanoparticle vaccines, viral vector vaccines, and nucleic acid vaccine technology. Every strategy offers distinct benefits and innovations that improve vaccine development processes' safety, effectiveness, and speed **(**Fig. 4**)**. In this section, authors have discussed selective technologies.

### Genomic sequencing

Determining the order of nucleic acid residues in biological samples is essential for various scientific purposes. Over the past 50 years, much research has gone into developing the techniques and instruments required to sequence DNA and RNA molecules [97]. Lucy van et al. study noted that genomic sequence use in the COVID-19 pandemic not only offered previously unheard-of insights into the historical demography of SARS-CoV-2 but also tracked the virus's adaptation to its new human host, providing information that informs the development of drugs and vaccines [98]. Genomic data can be utilized to characterize the more evolutionarily constrained sections of a pathogen genome that should be targeted preferentially to prevent rapid drug and vaccine escape mutants and uncover pathogen genes interacting with the host [99]. Furthermore, a nomenclature system can help with real-time epidemiology in an ongoing and dynamic epidemic like SARS-CoV-2 by offering globally accepted labels for circulating viruses. This helps identify the connections between outbreaks that have similar virus genomes [100]. The various mutations found at each location on the genome and the mutations assumed to occur are displayed by genomic sequencing. The distribution of the segregating variant in the phylogeny and on the map can be found by choosing a site in the genome with non-zero entropy. This makes it possible to investigate genetic alterations that could be adaptive or the root cause of altered disease dynamics [101].

A study by Robert P. et al. revealed that distinct viral variants, as determined by genomic sequencing, exhibit varying receptor attachment. Specifically, the mutants exhibit a significant decrease in binding to avian-type (α2–3) receptors and an increase in binding to human-type (α2–6) receptors. This makes it possible for various therapeutic and vaccination biomedical researchers to develop unique strategies [102]. Several full or partial Zika virus genomes, primarily from Brazil, and presenting information produced by a mobile genomics lab revealed that, According to estimates produced by combining analyses of viral genomes with ecological and epidemiological data, the Zika virus was likely already prevalent in northeast Brazil by February 2014. It had spread throughout the country and beyond before it was first discovered in the Americas. According to a study about establishing and transmitting the Zika virus in Brazil and the Americas, estimated dates for the virus's international spread from Brazil reflect the length of pre-detection cryptic transmission in recipient regions [103].

The genetic makeup of a virus can be ascertained using genome sequencing, which can also be used to create vaccinations that target particular proteins or genomic regions. The COVID-19 vaccines, which target the virus's spike protein, are developed using this technology. The genetic makeup of a virus can be determined using genome sequencing, which can also be used to create vaccines that target particular proteins or genomic areas. Furthermore, despite efforts to prevent it, SARS-CoV-2 sequencing has proven crucial in tracking mutations during the COVID-19 pandemic and understanding how the virus spreads to and from patients and healthcare workers [104]. Governments utilize the genetic laboratories' data to monitor the epidemic's spread, organize public health campaigns, and ensure that patients can get drugs and vaccines. In a study by Stefan Elbe et al., this was emphasized as extremely important. They also highlighted the likelihood of a potentially disastrous human pandemic and the comparatively rapid evolution of influenza viruses [105].

The table below **(**Table 2**)** delivers different summarized descriptions of genomic sequencing technologies used to gain information about the different microorganisms like Coronavirus, Ebola virus, etc. The information acquired is used to develop different prophylactic and therapeutic vaccines against those organisms.

**Table 2:** Uses of genomic sequencing technologies

**Serial No**	**Detailed use of genomic sequencing technology**	**viruses**	**Vaccine**	**Reference**
1	Tracks the virus’ adaptation to its new host and gives an over view of the genome	Corona virus	Pfizer-BioNTech and Moderna vaccines	[98, 100, 106]
2	Gives a view about the spread of the disease	Ebola virus	rVSV-ZEBOV vaccine	[101, 107]
5	Notification about the different mutations of a given virus, use of the different receptors in the body after mutation	Avian Influenza, Human H1 and H3N2 viruses	mRNAseasonal Influenza vaccine	[102, 108]
6	Identification of the origin of the virus genome before and after mutation	Zika virus	VRC5288 and VRC5283 Vaccines	[103, 109]
7	Genetic information about the virus spread from patients to health workers	Corona virus	NVX-CoV2373 vaccine	[110, 111]
8	Control epidemics from becoming pandemics	Influenza virus	CRISPR –Cas systems influenza vaccines	[105, 112]

### Nucleic acid vaccine technology

Nucleic acid vaccines are novel vaccinations that teach cells to generate an antigen that elicits an immune response using genetic material, either DNA or RNA. The nucleic acids are expressed after being ingested by antigen-presenting cells. They are non-infectious but imitate natural infection by producing endogenous antigens and evoking potent T and B cell responses [106].

#### Messenger ribonucleic acid (mRNA) vaccine technology

One significant scientific advance in recent decades has been the creation of mRNA treatments and vaccinations. Researchers like Katalin Karikó and Drew Weissman first proposed employing mRNA as a therapeutic tool in the early 1990s when they found that synthetic mRNA might be altered to lessen its intrinsic immunogenicity [113]. This was essential since unmodified mRNA could cause an undesired immune response and was easily degraded. They improved mRNA's translational efficiency and stability by introducing modified nucleosides, which set the stage for its application in vaccines and treatments. Targeting the rabies virus, the first mRNA vaccine to reach clinical trials was created in 2013, showcasing the technology's potential for use in people. However, the COVID-19 pandemic of 2020, which led to the quick creation, licensing, and widespread distribution of Moderna's mRNA-1273 and Pfizer-BioNTech's BNT162b2 vaccines, was what brought attention to the benefits of mRNA vaccines [114]. These vaccines demonstrated the versatility of mRNA technology in treating newly emerging infectious diseases in addition to their speed and scalability. A new era in vaccination research has been ushered in by the success of these vaccines, which have prompted additional studies into mRNA-based treatments, including ones that target infectious diseases and cancer [115].

Katalin Karikó et al. revealed that because mRNA is non-infectious and degrades naturally by cellular processes, it has been an asset in vaccine creation through research and various technologies over the years. Moreover, mRNA is much more translatable and stable [116]. The production of mRNA involves in vitro reactions with recombinant enzymes, ribonucleotide triphosphates (NTPs), and a DNA template, as reported in a study by Norbert P et al. This makes mRNA production faster and more accessible than conventional protein subunits and live or inactivated vaccine production platforms [117]. When a vaccine is encapsulated in lipid nanoparticles (LNPs) for optimal cellular uptake and protection, the mRNA stays in the host cell's cytoplasm [118]. The antigen protein is produced by the host cell's machinery using genetic instructions. On the cell surface, the antigen is shown, triggering the humoral and cellular immune responses and forming memory cells, which offer long-term immunity [119].

Multiple vaccine candidates against COVID-19 are included in the promising class of mRNA-based vaccines. A nucleoside-modified mRNA-lipid nanoparticle (mRNA-LNP) vaccine that encodes the receptor-binding domain of the spike protein as a monomer or the whole SARS-CoV-2 spike glycoprotein [120, 121]. Because of its safety, uncomplicated design, and ease of manufacture, mRNA research has continued. Finally, tenacity paid off, as proven by the discovery of highly effective COVID-19 mRNA vaccines, which have played a critical role in continuing pandemic control efforts [122]. The mRNA vaccines used currently are mRNA-1273, developed by Moderna, and BNT162b2, developed by Pfizer-BioNTech [106]. Monslow et al. tested the efficacy of an LNP-enclosed mRNA vaccine encoding the varicella-zoster virus (VZV)gE antigen against two other approved vaccines. The results showed that the VZV gE mRNA/LNP platform can elicit a robust immune response [123].

**Fig. 5:**
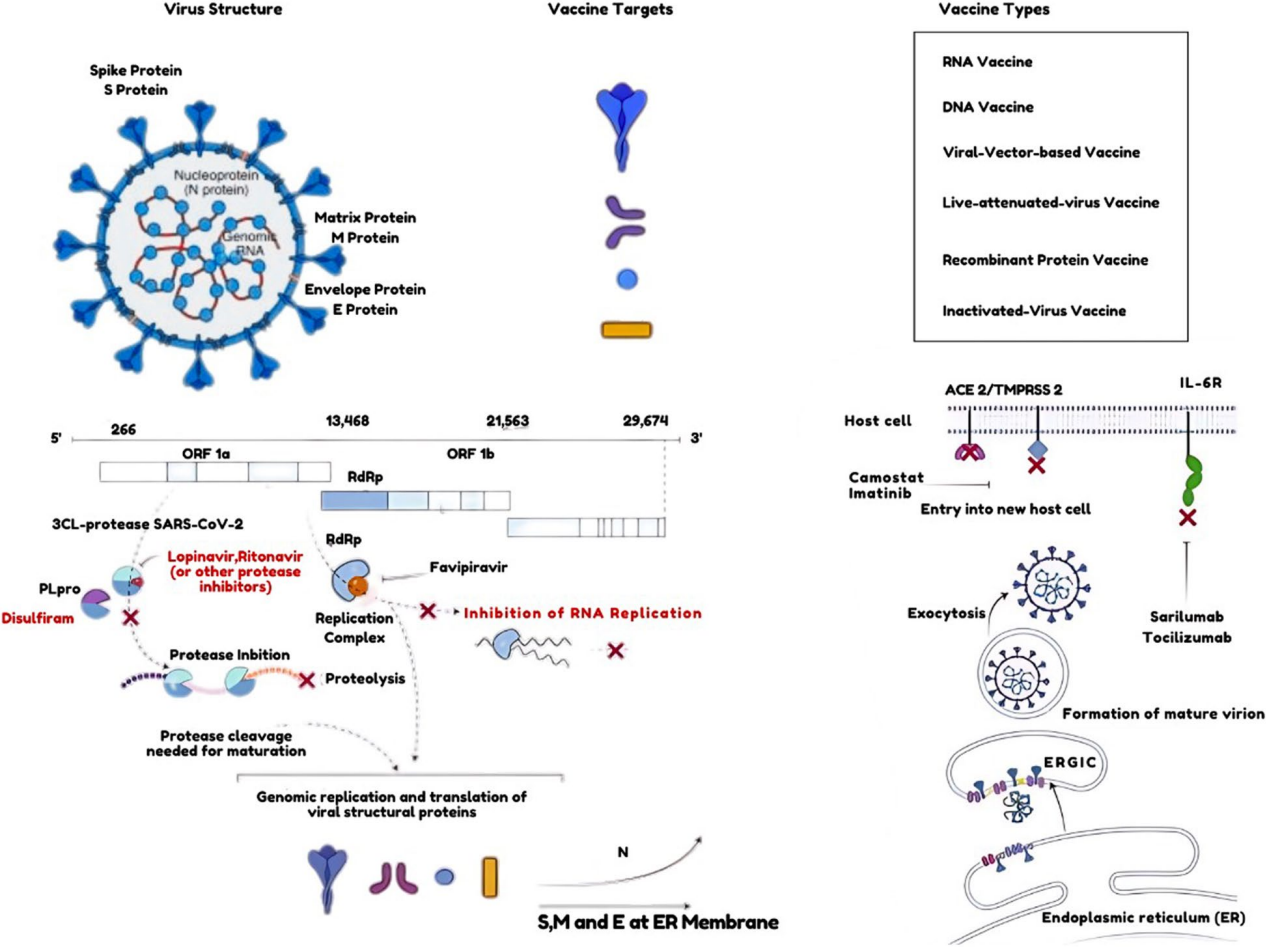
Schematic representation of techniques for Treatment and Prevention against SARS-Cov-2. Techniques for Treatment and Prevention Against SARS-Cov-2.a), Vaccine targets and vaccine platforms against SArS-coV-2. b, examples of viral targets and of potential antiviral drugs against SArS-coV-2. OrF, open reading frame; rdrp, rNA-dependent rNA polymerase; PLpro, papain-like protease; Ace 2, angiotensin-converting enzyme 2; TmPrSS 2, transmembrane protease, serine 2; er, endoplasmic reticulum; erGIc, endoplasmic-reticulum–Golgi intermediate compartment; IL-6r, interleukin-6 recseptor

A study by Justin M. et al. revealed that since there is now no medication to combat the Zika virus, immunization is the only way to avoid the risks of infection. mRNA vaccines against the Zika virus commonly use membrane and envelope proteins as antigens [124]. Influenza viruses are of four strains: A, B, C, and D; however, types A and B are clinically linked to human diseases. According to a study by Benjamin P. et al., an mRNA vaccine was developed against the human influenza virus; the target of the vaccine is the glycoprotein hemagglutinin (HA) on the virus's surface since it mediates the viral entry [125]. A paper published by Kai Wu et al. described in detail the immunological responses that both seropositive and seronegative healthy volunteers from a phase 1 randomized, first-in-human clinical trial had in response to the investigational cytomegalovirus (CMV) vaccine, mRNA-1647. The results of the investigation showed that both fibroblasts and epithelial cells responded strongly and broadly to three dosages of mRNA-1647 (180 μg) when it came to CMV infection [126, 127]. Figure 5 shows the techniques for treatment and prevention against SARS-Cov-2.

According to findings from a study by Mia et al., cellular immune responses to the rabies mRNA vaccine RV021 at 5 μg may be more powerful than those to Inactivated Rabies Vaccines (IRVs). The cellular immune response was significantly improved by switching from one dose of RV021 to two doses, as opposed to increasing the single dose from 1 μg to 5 μg [128]. For many years, there have been unsuccessful attempts to create effective anti-HIV vaccinations. The new mRNA vaccine technique has made great strides recently in terms of adoption as an anti-HIV strategy. Several mRNA-based HIV vaccines are being tested in clinical settings to determine their efficacy and safety [129].

A revolutionary development in immunization, mRNA vaccine technology has several distinct benefits over conventional vaccination platforms. Instead of growing live viruses, these vaccines can elicit a potent immune response by using synthetic mRNA to tell cells to make viral proteins. This creative method has sped up the production and distribution of vaccines, as the COVID-19 pandemic has shown. Nevertheless, mRNA vaccines have their own set of difficulties and restrictions, much like any new technology, particularly in contrast to the more well-established conventional vaccinations. Table 3 offers each platform's benefits and drawbacks by comparing mRNA vaccines and conventional vaccinations in several areas.

**Table 3:** The advantages and disadvantages of each platform, including mRNA vaccines and traditional vaccines

**Sr.No**	**Aspects**	**Advantages**		**Disadvantages**		**References**
		**mRNA Vaccines**	**Traditional Vaccines**	**mRNA Vaccines**	**Traditional Vaccines**	
1	Development Speed	Rapid design and production do not require virus cultivation	Established processes with predictable timelines	Limited long-term data on safety and efficacy	Requires the cultivation of live viruses or proteins, which takes more time	[115, 130]
2	Flexibility	Easily updated for new variants by modifying the mRNA sequence	Can be updated for new strains	Requires ultra-cold storage, complicating distribution	Slower adaptation to rapidly mutating pathogens	[131]
3	Immune Response	Induces robust antibody and T-cell responses	Provide long-lasting immunity with a single dose or a few doses	The durability of immunity is still under study	May require multiple doses or boosters to maintain immunity	[132]
4	Storage and Stability	None	Generally stored at 2–8 °C, suitable for global distribution	Ultra-cold storage (-70 °C) is required for some, limiting use in low-resource settings	Live-attenuated vaccines need precise storage to maintain potency	[133]
5	Delivery Mechanism	LNPs effectively deliver mRNA into cells for protein production	Includes injections, oral vaccines, nasal sprays, etc	NPs can cause mild side effects	Live-attenuated vaccines may cause more pronounced side effects	[134, 135]
6	Long-Term Data	None	The long history of use	Ongoing collection of long-term safety and efficacy data	May not be as effective against new or rapidly mutating pathogens	[132]
7	Production Scalability	High scalability	Scalable	Requires advanced manufacturing facilities and strict quality control	Requires specialized facilities for the growth and inactivation of pathogens	[131]
8	Global Accessibility	Potentially high	Widely accessible	Logistically challenging	Variable	[117]
9	Regulatory Approval	Accelerated approval	Established pathways	Rigorous oversight	Time consuming	[132]
10	Public Perception	Innovative	Trusted	Skepticism	Vaccine hesitancy	[136]
11	Customizability	Highly customizable	Established methods	Complex design	Limited flexibility	[137]
12	Efficacy Across Populations	Consistent efficacy	Proven efficacy	Uncertain long-term efficacy	Variable response	[132]
13	Side Effects and Tolerability	Mild side effects	Well-known profile	Unknown rare effects	More severe side effects	[138]
14	Targeted Delivery	Precision targeting	General efficacy	Delivery challenges	Less specific	[139]
15	Pandemic Preparedness	Rapid deployment	Historical success	Dependent on advanced infrastructure	Slower response	[140]

##### Therapeutic mRNA vaccines

In contrast to a preventative strategy for infectious diseases, mRNA vaccines are typically used therapeutically to treat cancer [141]. Its usual purpose is to encrypt neoantigens or tumor-associated antigens (TAAs) in order to trigger antitumor immune responses as used in cancers such as melanoma, brain, ovarian, prostate, blood system, digestive system, and breast cancers, as well as non-small cell lung cancer [116]. The malignant transformation of melanocytes, which are found throughout the body, including the skin, mucosa, uvea, inner ear, and rectum, is the cause of melanoma. The primary use for DC-based mRNA vaccines is against melanoma, which induces a broad spectrum of T-cell responses when injected [142].

Additionally, a study by Yanina J et al. revealed that in patients with metastatic melanoma, autologous monocyte-derived mRNA co-electroporated dendritic cells expressing mRNA encoding CD40 ligand (CD40L), CD70, and constitutively activated TLR4 (caTLR4) exhibit antitumor efficacy. These cells are known as TriMixDC-MEL. Adjuvant therapy with TriMixDC-MEL id/iv is acceptable and can potentially increase the 1-year disease-free survival rate [143]. The most prevalent primary brain tumor in adults, glioblastoma (GBM), has a median survival of fewer than two years when treated with standard care [144]. It is always fatal. Immunotherapies based on RNA offer a significant chance of establishing a long-lasting therapeutic response for GBM and other malignant brain tumors. Although RNA has apparent benefits over antigen-focused strategies, its biological instability frequently prevents it from being delivered directly, so it is usually used in RNA dendritic cell vaccinations and RNA nanoparticle treatments [145].

Globally, lung cancer is the primary cause of cancer death and the second most common type of cancer. Non-small cell lung cancer by mRNA 85% of all lung cancers are non-small cell lung cancers (NSCLCs) [146]. Two mRNA-based vaccines, CV9201 and BI1361849 (CV9202), were clinically studied in NSCLC. A study by Martin Sebastian et al. demonstrated that CV9201 is an RNActive-based cancer immunotherapy encoding five NSCLC antigens [147]. Ovarian cancer is the fifth most significant cause of cancer-related deaths in women worldwide, accounting for less than 5% of all cancer-related deaths in women [148]. Tetiana et al.'s study demonstrated the use of lipid nanoparticles to transfer mRNA encoding the follistatin protein. This protein inhibits activin A, a protein linked to both cachexia, a disease where patients with cancer frequently experience muscle atrophy, and aggressive ovarian cancer [149].

According to Global Cancer Statistics 2020, breast cancer in women has overtaken lung cancer as the most common disease to be diagnosed. Triple-negative breast cancer (TNBC), HER2 + , and ER + are the three main subtypes of breast cancer [148]. Among the various subtypes of breast cancer, the mutational burden is highest in TNBC. Furthermore, increased tumor-associated antigen levels imply that immunotherapies, particularly for TNBC, are a promising therapeutic alternative [150]. According to recent research, mRNA vaccines have more immunostimulatory antigens than peptide-loading DCs and vaccinations based on HER2 + tumor cells. They thus produced the greatest cell lysis by the in vitro HER2-specific cytotoxic T cell (CTL) induction [151].

There have been reports that mRNA vaccines could be used to cure a variety of uncommon illnesses. For example, cystic fibrosis is a genetic disorder affecting the lungs, pancreas, and other organs [152]. Robinson et al. demonstrated a promising platform for the correction of cystic fibrosis. They found that a chemically modified mRNA encoding Cystic Fibrosis Transmembrane Conductance Regulator (CFTR), packed in lipid nanoparticles, increased membrane-localized CFTR and preserved its function as a chloride channel in patient-derived bronchial epithelial cells. When applied topically, the modified mRNA restored CFTR-mediated chloride secretion to conductive airway epithelia in CFTR-deficient mice [36].

##### Prophylactic mRNA vaccines

mRNA is much more translatable and stable [116]. The production of mRNA involves in vitro reactions with recombinant enzymes, ribo NTPs, and a DNA template, as reported in a study by Norbert P et al. This makes mRNA production faster and more accessible than conventional protein subunit and live or inactivated vaccine production platforms [117]. When a vaccine is encapsulated in LNPs for optimal cellular uptake and protection, the mRNA stays in the host cell's cytoplasm [118]. The antigen protein is produced by the host cell's machinery using genetic instructions. On the cell surface, the antigen is shown, triggering the humoral and cellular immune responses and forming memory cells, which offer long-term immunity [119, 153].

Multiple vaccine candidates against COVID-19 are included in the promising class of mRNA-based vaccines. A nucleoside-modified mRNA-LNP vaccine that encodes the receptor-binding domain of the spike protein as a monomer or the whole SARS-CoV-2 spike glycoprotein [120, 121]. Because of its safety, uncomplicated design, and ease of manufacture, mRNA research has continued. Finally, tenacity paid off, as proven by the discovery of highly effective COVID-19 mRNA vaccines, which have played a critical role in continuing pandemic control efforts [122]. The mRNA vaccines used currently are mRNA-1273, developed by Moderna, and BNT162b2, developed by Pfizer-BioNTech [106].

Monslow et al. tested the efficacy of an LNP-enclosed mRNA vaccine encoding the varicella-zoster virus (VZV) gE antigen against two other approved vaccines. The results showed that the VZV gE mRNA/LNP platform can elicit a robust immune response [123]. A study by Justin M. et al. revealed that since there is now no medication to combat the Zika virus, immunization is the only way to avoid the risks of infection. mRNA vaccines against the Zika virus commonly use membrane and envelope proteins as antigens [124]. Influenza viruses are of four strains: A, B, C, and D; however, types A and B are clinically linked to human diseases. According to a study by Benjamin P. et al., an mRNA vaccine was developed against the human influenza virus; the target of the vaccine is the glycoprotein HA on the virus's surface since it mediates the viral entry [125].

A paper published by Kai Wu et al. described in detail the immunological responses of seropositive and seronegative healthy volunteers from a phase 1 randomized, first-in-human clinical trial in response to the investigational CMV vaccine, mRNA-1647. The investigation showed that both fibroblasts and epithelial cells responded strongly and broadly to three dosages of mRNA-1647 (180 μg) regarding CMV infection [126].

By encoding for a stable prefusion F glycoprotein, which is essential for the virus to infect host cells, this experimental vaccination specifically targets the Respiratory Syncytial Virus (RSV). Results from clinical trials have been encouraging, especially for older persons. A vaccination efficacy of 83.7% against RSV-associated lower respiratory tract disease with two or more symptoms was shown in Phase 3 ConquerRSV research. The FDA has designated Moderna's mRNA-1345 as a Breakthrough Therapy [127].

#### DNA vaccines

**Fig. 6:**
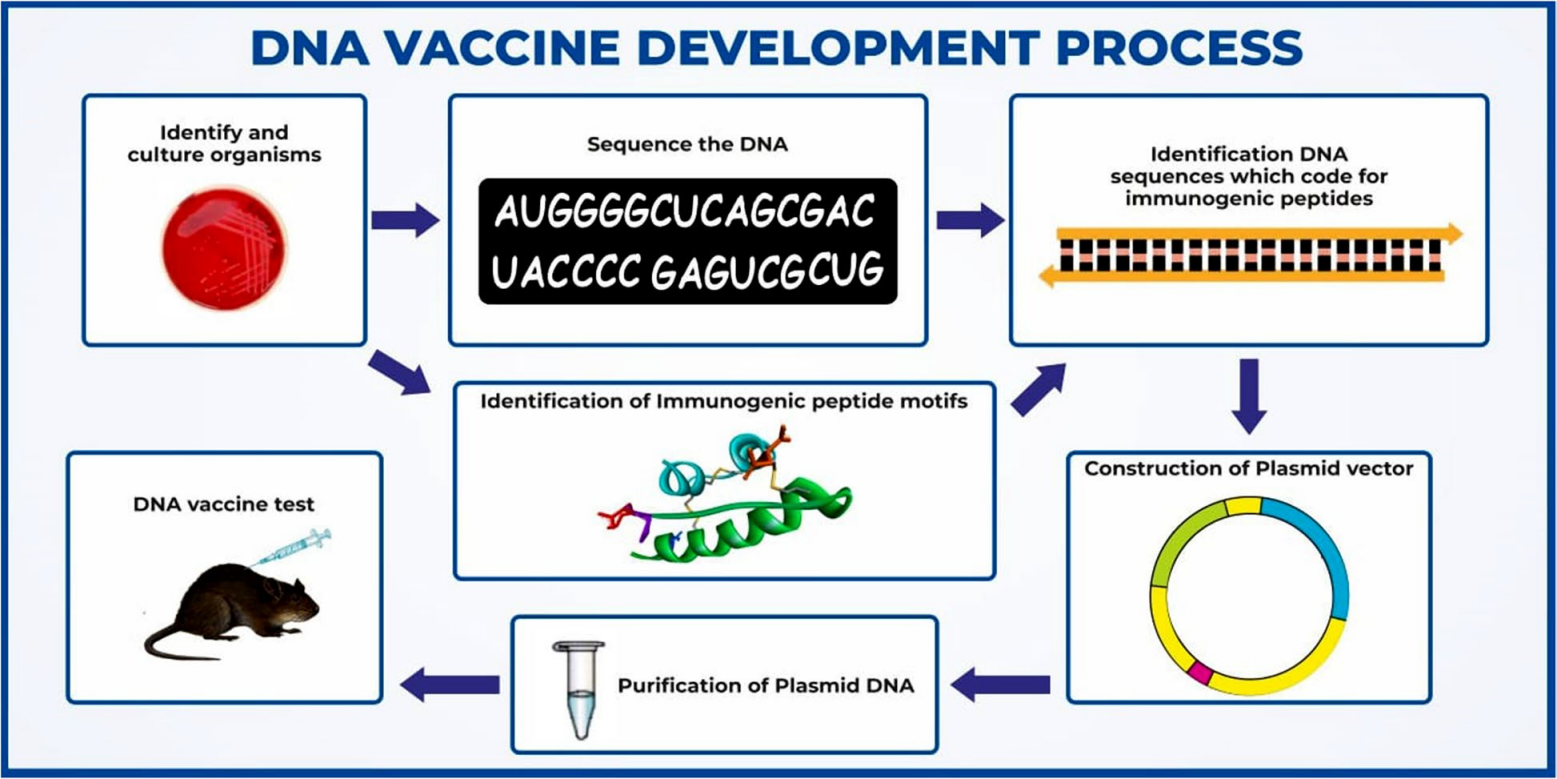
The DNA vaccine development process, starting with identifying and culturing organisms, sequencing their DNA, and identifying immunogenic DNA sequences. These sequences are used to identify immunogenic peptide motifs, which are then inserted into a plasmid vector. The plasmid DNA is purified and tested as a DNA vaccine in animal models

Direct injections of genetically modified DNA encoding the antigen are used in DNA vaccines. The host cells take up the DNA, creating the antigen and triggering an immunological reaction. DNA vaccines have shown promise in preclinical and clinical trials while still primarily experimental [154]. Using recombinant DNA technology, antigens that can be included in vaccinations are created by modifying DNA sequences. DNA vaccines differ from traditional protein or peptide vaccinations, recombinant proteins, or virus-like particles in that they typically elicit robust humoral and cellular (Th1 type CD4 + T cells and CD8 + cytotoxic T cells) responses upon in vivo introduction of genetic material [155]. Developing a DNA vaccine starts with identifying and culture the intended target organisms. The genetic sequences that encode immunogenic peptides are then found by sequencing the DNA of these organisms. The ensuing identification of immunogenic peptide motifs depends on these sequences. Following their identification, these motifs are put into a plasmid vector, which is subsequently built to include the genetic material required for the immunological response. After purification, the plasmid DNA is ready for examination. Lastly, tests of its safety and effectiveness are conducted on animal models to confirm the DNA vaccine's ability to elicit the intended immune response **(**Fig. 6**).**

##### Prophylactic recombinant DNA technology vaccines

Cervical cancer and other malignant tumors are known to be primarily caused by persistent HPV infection. Surgical treatments and efficient HPV screening technologies can help diagnose and cure cervical cancer early on. As a more potent remedy, vaccinations are anticipated to avert viral infection and the ensuing illnesses during the prophylactic and curative stages [156]. Global licensing has been granted for three prophylactic HPV vaccinations: quadrivalent, bivalent, and nonavalent [157]. A study by Stanley et al. revealed that each vaccine consists of virus-like particles (VLPs) with an L1 main capsid protein that has been recombinant produced from a particular type of HPV. All of them focus on high-risk HPV strains 16 and 18, which account for over 71% of occurrences of cervical cancer globally [158].

Furthermore, a study by Rashi Y et al. found that the most recent HPV vaccine, Gardasil-9 (a nonavalent vaccine), offers minimal cross-protection against non-vaccine HPV types and protects against seven HPV types linked to about 90% of cervical cancer cases as well as two HPV types linked to about 90% of genital wart cases [159]. Hepatitis B recombinant vaccine, the noninfectious subunit hepatitis B vaccine Engerix B((R)) [Hep-B(Eng)], is recommended for the active immunization of adults, children, and newborns against hepatitis B virus infection. It comprises the hepatitis B surface antigen (HBsAg), created via recombinant DNA technology by the yeast Saccharomyces cerevisiae [160].

The creation of a DNA influenza vaccine is now a more realistic objective. A single consensus H5 antigen was used to produce protective antibody titers to several clades of H5N1 in a preclinical investigation of an H5N1 influenza DNA vaccine [161]. Furthermore, their preclinical model success has made the development of DNA vaccines against multiple influenza strains possible. Several DNA-based influenza vaccines, including those against potentially deadly pandemic strains like H5N1 and H1N1, are in various phase I clinical trials [162]. When administered alone or in conjunction with aluminum-based adjuvants, inactivated split-virus vaccines have shown some degree of immunogenicity after two doses spaced 21–28 days apart [163].

Several methods are being researched to increase the immunogenicity of potential HIV-1 DNA vaccines. The expression of many antigens, safety, and ease of design make DNA vaccines advantageous. A study by Srilatha et al. did a clinical trial on the PENNVAX®-GP DNA vaccine was evaluated in the HVTN 098 trial in healthy adult participants at 1-,3-, and 6-month intervals via intradermal (ID) or intramuscular (IM) electroporation (EP). The trial reported positively on safety, tolerability, and acceptability [164].

##### Therapeutic recombinant DNA technology vaccines

The development of HPV vaccinations for patients with established CIN2/3 and invasive cervical cancer has been the focus of ongoing research efforts; for patients with HPV16-associated CIN2/3, a study by Alvarez et al. showed the viability, safety, and possible therapeutic efficacy of pNGVL4a-CRT/E7(detox), a new DNA vaccine made of a pNGVL4a plasmid vector harboring a mutant version of the HPV16 E7 antigen linked to calreticulin (CRT) [165]. Using therapeutic DNA vaccines against mycobacterium TB is a promising strategy, according to a study by Changhong et al. IL-2 and the HSP65 fusion gene expression in DNA vaccines were investigated. It improved the HSP65-DNA vaccine's immunogenicity, therapeutic benefits, and protective qualities against tuberculosis in mice. By enhancing the Th1-type response, this was accomplished [166].

### CRISPR-Cas systems

Programmable endonucleases found in microbial CRISPR-associated (CRISPR-Cas) and Clustered Regularly Interspaced Short Palindromic Repeats (CRISPR) systems can be used for CRISPR-based diagnostics (CRISPRDx) [167]. Gene-editing techniques called CRISPR-Cas systems have recently been modified for application in antiviral treatments, including HIV, Influenza, and SARS-CoV-2. By focusing on particular viral genes and impairing their function, CRISPR-based treatments can stop the virus from proliferating. These therapies work by concentrating on viral proteins that are conserved across all viruses [168].

Mireille Be'termier et al.'s study revealed that by causing a double-strand break (DSB) at a target genomic locus, CRISPR-Cas9 systems facilitate genome editing [169]. The cell's DNA repair mechanism promptly repairs the DSB. The technique known as non-homologous end joining (NHEJ), which is highly accurate, can be used to link the ends of DNA that are created. Because of its superior simplicity, efficiency, and multiplex ability compared to other nucleases like zinc finger nucleases (ZFNs) and transcription-activator-like effector nucleases (TALENs), CRISPR-Cas9 technology has become the most widely used method for genome engineering available today [170].

#### Prophylactic technology

A study by Kaito A. et al. noted that with the ability to knock out or modify specific genes essential for viral virulence, CRISPR/Cas technology has emerged as a revolutionary tool in genetic engineering with great potential for creating influenza vaccines [112]. The result is a virus that is still immunogenic but not pathogenic.HBV treatment entails inactivating HBV cDNA, which is difficult because of its placement within the nucleus. To overcome this issue, gene editing can be used with gene-silencing technologies as a potential anti-HBV therapy [171].

#### Therapeutic vaccines technology

It has been demonstrated that employing multiplexed guide RNAs (gRNAs) in conjunction with CRISPR/Cas9 to cleave multiple HBV genome areas at once increases the efficiency of HBV genome ablation and elimination. Completely removing a full-length 3175 bp HBV DNA from the host genome using CRISPR/Cas9 technology [172]. The CRISPR/Cas9 system can precisely target and cleave conserved areas in the HBV genome, leading to potent inhibition of viral gene expression and replication, according to a study by Vyas Ramanan et al., adding that this might be a cutting-edge therapeutic strategy to manage the virus and perhaps even heal people [173].

### Viral vector vaccines

Vaccines with viral vectors introduce genetic material into cells through altered viruses, inducing an immunological response. To increase safety, these vectors can be less capable of replicating. Vaccines based on viral vectors come in two varieties: replicating and non-replicating. Replication-deficient viral vectors are used in non-replicating viral vector-based vaccinations to transfer a specific antigen's genetic material to the host cell and create immunity against the target antigen [79].

#### Prophylactic viral vector vaccines

The JNJ-78435735 vaccine from Johnson and Johnson (Janssen) and Beth Israel Deaconess Medical Center, AZD1222 from Oxford-AstraZeneca, Sputnik V and Sputnik Light from Gamaleya Research Institute of Epidemiology and Microbiology, and Convidecia vaccine from CanSino Biologics are the current adenovirus vector-based vaccines being used to prevent SARS-CoV-2 infection [174]. Furthermore, the FDA in the US has authorized the Janssen vaccine for use in emergencies out of the five vaccinations [175]. Once injected into the host cell, the genetically altered adenovirus 26 is transformed into a vaccine absorbed by the cell [176].

The AZD1222 vaccine, formerly ChAdOx1 nCoV-19, is also called Covishield, Vaxzevria, Oxford-AstraZeneca Vaccine, and others [174]. It proved effective and safe in preventing severe and symptomatic Covid-19 in various demographics, including senior citizens. According to the phase 3 study, the vaccine had a 76% efficacy rate in avoiding symptomatic COVID-19 infection and a 100% efficacy rate in preventing hospitalizations and severe or critical illness [177].Gam-COVID-Vac trade-named Sputnik V, so named after the first artificial satellite. It is an adenoviral vector-based COVID-19 vaccine developed by the Gamaleya Research Institute of Epidemiology and Microbiology. The vaccine was designed with two recombinant adenovirus vectors and was developed as two formulations (frozen [Gam-COVID-Vac] and lyophilized [Gam-COVID-Vac-Lyo [178]. Random mutations and deletions brought about by the passage of parental VACA contributed to VACV's decreased pathogenicity. Listeria clone 16m8 (LC16m8), Dairen I strain (Dis), M65, M101, modified vaccinia virus Ankara (MVA), and many attenuated fowl pox viruses are among the vectors of the third generation of poxviruses [179].

#### Therapeutic viral vector vaccines

Vaccines with viral vectors are utilized more often to treat cancer because of their potent immune-stimulating effects. These vaccinations encourage the immune system to recognize and eliminate cancer cells [180]. A study by Jei Wang et al. revealed that an Oncolytic adenovirus is a novel form of viral preparation with low cytotoxicity and good selectivity for treating castration-resistant prostate cancer (CRPC) [181]. Prostate cancer cells can undergo apoptosis when exposed to the oncolytic mutant Ad deleted without E1B19K and E1ACR2 [182].

Furthermore, the recombinant vaccinia virus, which can trigger the first immune response, and the fowl pox virus, which is utilized to boost the immune response, are the two components of Prostvac, a tumor vaccine based on recombinant poxvirus vector [183]. Viral vector vaccines are employed in gene therapy to replace damaged genes with functional ones, hence treating genetic diseases. The most well-known viral-based vectors for gene delivery are lentivirus, adenovirus, and adeno-associated virus (AAV) [184]. A study by Prado et al. revealed that The FDA has approved Luxturna as a gene therapy for Leber's congenital amaurosis, a hereditary condition that results in blindness. The RPE65 gene is functionally delivered to retinal cells via an AAV vector. The therapy restores vision for those suffering from this uncommon hereditary condition [185].

### Protein subunit vaccines

The biotechnology field is expanding quickly, allowing for further advancements in the investigation of antigen suitability as potential vaccination candidates. Protein subunit vaccinations expose the immune system to one or more antigens, often presented as isolated or purified proteins, polysaccharides, nucleic acids, or specific portions of proteins that have been shown to have immunogenic qualities [186].

The conjugate vaccine for Haemophilus influenzae type b (Hib), which consists of a polysaccharide-protein conjugate, is an example of a successful subunit vaccination. According to research by Muganga et al. conducted in Rwanda, this vaccination has eradicated or significantly decreased this disease in children in regions of Africa [187]. The European Medicines Agency (EMA) has given its reasonable scientific opinion to RTS, S/AS01, the first and only malaria vaccine. In order to boost the immune response, it targets the circumsporozoite protein (CSP) of Plasmodium falciparum in conjunction with the adjuvant AS01. With roughly 39% efficacy in preventing malaria in young children and 29% efficacy in babies, the vaccination offers only modest protection against the disease [188].

According to a study by Koupaei M, immunological responses are elicited by protein subunits that contain a particular viral product rather than the entire viral particle. Structural and non-structural proteins are present in SARS-CoV-2 in addition to accessory proteins. The primary SARS-CoV-2 structural proteins are membrane (M), envelope (E), and (S) proteins [189].

### Nanoparticle vaccines

The use of nanoparticle-based vaccinations has drawn much attention in recent years to enhance immunization tactics, vaccine efficacy, and targeted delivery to produce the intended immune responses at the cellular level [149]. When developing vaccines, NPs can also be designed to make it easier for antigens to be included. NPs can include antigens through encapsulation (physical trapping) or conjugation (covalent functionalization). Because antigenic material is encapsulated in NPs, antigens that would otherwise rapidly degrade or elicit a localized immune response can be administered [190]. The effectiveness of the COVID-19 mRNA vaccines demonstrates the potential of nanoscale platforms as safe and efficient vaccine delivery systems. Additionally, native pathogen-associated molecular patterns (PAMPs) found in biologically generated nanoparticles can lessen the requirement for synthetic adjuvants [191].

**Fig. 7:**
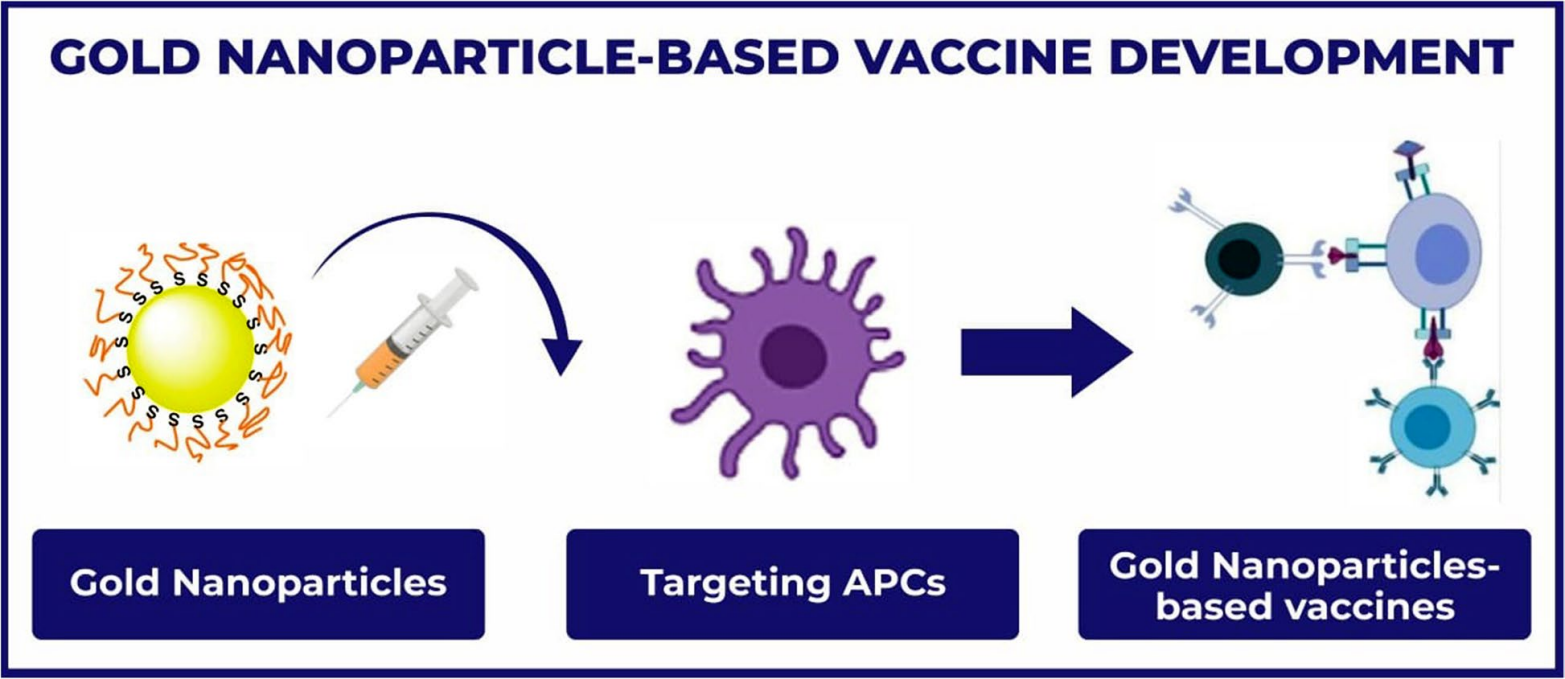
Vaccine development process using gold nanoparticles

*Gardasil* is a four-component vaccination that targets HPV specifically and is based on VLPs. It is given with an aluminum adjuvant and contains the L1 main capsid protein of HPV types 6, 11, 16, and 18. The HPV combination vaccine was 100% effective in preventing clinical illness and was immunogenic, producing long-lasting antibodies, as noted in a study by Villa et al. [192]. Three trials have used the Respiratory Syncytial Virus Fusion (RSVF) nanoparticle vaccination with aluminum in phases two and three to prevent RSV infections. The RSVF Dose-Ranging Study in Women was the first clinical study of RSV, lasting from October 2013 to May 2016. According to the study, no significant adverse effects were associated with the medication, and all formulations were well tolerated [193]. Figure 7 presented the development of gold nanoparticle-based vaccines, where gold nanoparticles target APCs to enhance the immune response.

Ferritin-backed self-assembling nanoparticle vaccine candidates may be produced practically and safely, and this platform offers unique benefits in terms of flexible assembly and comparatively easy scale-up. Heng Li et colleagues. used rhesus monkeys to explore the immune responses and protective impact of Receptor-Binding Domain (RBD) ferritin nanoparticles and to develop a SARS-CoV-2 vaccination platform with strong immunogenicity, high protection efficiency, broad range, and good safety [194]. Table 4 shows the various biomedical technologies and innovations that have contributed to the development of vaccines, with examples.

**Table 4:** Various biomedical technologies and innovations that have contributed to the development of vaccines with examples

**S/N**	**Technology**	**Use**	**Example**	**Reference**
1	mRNA	Prophylactic	1273 (Moderna), BNT162b2 (Pfizer-BioNTech) COVID-19 vaccines	[106, 122]
2	mRNA	prophylactic	Varicella zoster vaccine (vzvgE)	[123]
3	mRNA	Prophylactic	Zika virus vaccine	[124, 195]
4	mRNA	Prophylactic	Influenza Vaccine	[125]
5	mRNA	Prophylactic	Cytomegalo virus vaccine	[126]
6	mRNA	Prophylactic	RSV	[127]
7	mRNA	Prophylactic	Rabies virus vaccine	[128]
8	mRNA	Prophylactic (clinical trials)	HIV vaccine	[129]
9	mRNA	Therapeutic	Melanoma vaccines	[142, 143, 196]
10	mRNA	Therapeutic	Glioblastoma vaccines	[145]
12	mRNA	Therapeutics	NSCLC	[147]
13	mRNA	Therapeutics	Ovarian cancer	[149]
14	mRNA	Therapeutics	Breast cancer	[150, 151]
15	mRNA	Therapeutics	Cystic fibrosis	[152]
17	Recombinant DNA	Prophylactic	Cervical cancer (HPV)	[158, 159]
18	Recombinant DNA	Therapeutic	Cervical cancer (HPV)	[165]
19	Recombinant DNA	Therapeutic	Tuberculosis (MTB)	[166]
20	Recombinant DNA	Prophylactic	Avian influenza	[161, 163]
21	Recombinant DNA	Prophylactic	HIV	[164]
22	Recombinant DNA	Prophylactic	Hip B	[160]
23	CRISPR –Cas systems	Therapeutics	HBV	[173]
24	CRISPR –Cas systems	Prophylactic	Influenza	[112]
25	Viral vector	Prophylactic	SARS-COV-2 (Janssen vaccine Sputnik vaccine, Covishield)	[174, 175, 177, 178]
26	Viral vector	Prophylactic	Pox virus	[179]
27	Protein subunit	Prophylactic	Haemophilus influenza type B (HiB)	[187]
28	Protein subunit	Prophylactic	Malaria	[188]
29	Protein subunit	Prophylactic	SARS-CoV-2	[189],
30	Nano particles	Prophylactic	Covid-19	[190]
31	Nano particles	Prophylactic	HPV	[192]

## Key challenges in vaccine development and deployment

Developing effective vaccines involves overcoming numerous challenges. This highlights a few challenges, including delivery methods, antigen identification, and transportation and storage logistics. These challenges are critical to ensuring the success of vaccination campaigns and improving public health outcomes.

### Vaccine delivery and administration

They create efficient delivery methods that guarantee the vaccine's durability, bioavailability, and appropriate immunological presentation. This may use viral vectors, liposomes, or nanoparticles [117]. The obstacles associated with manufacturing, stability, and immune response modification are unique to each delivery strategy [197]; according to a study by Rashmirekha P et al., there has been much focus on using vaccines based on nanoparticles to enhance vaccination efficacy, immunization tactics, and targeted delivery to produce the intended immune responses at the cellular level. These nanocarriers must prevent premature proteolytic destruction of the antigens, aid in antigen absorption and processing by antigen-presenting cells, regulate release, and be safe for human usage to increase vaccine efficacy [198]. The recombinant nanoparticle vaccine known as rSARS-CoV-2 was created by Novavax and produced by Emergent BioSolutions. It is made from the complete, wild-type SARS-CoV-2 spike glycoprotein, including the transmembrane domain, as noted in a study by C. Keech et al. [199].

According to a study by Kato, vaccinations based on NPs have several drawbacks. The cellular toxicity of nanoparticles and the requirement for an adjuvant to increase vaccination efficacy are two of the main drawbacks of using them in vaccine design. Additionally, the effectiveness of vaccines is significantly impacted by the size, shape, charge, and surface area of NPs [200].

### Antigen identification and selection

It is essential but complex to determine which antigen, or combination of antigens, can elicit a robust and defensive immune response. The ability of the antigen to activate the immune system to identify and combat the infection is a requirement [201]. A study by Anna et al. noted that the need for more knowledge about the correlates of immune protection is another challenge for researchers. This is because HIV-1 infection progresses in a complicated way, and the immune system is never able to completely eradicate the virus due to the reservoir of latently infected memory CD4 + T-cells [202]. The procedure frequently entails an in-depth analysis of the structure and functionality of the pathogen. It is finding viral surface proteins, such as the spike protein of SARS-CoV-2, that may be one way to combat viruses. However, developing new viruses proved extremely difficult because they had to be inexpensive and safe to employ [203].

Identifying adjuvants that are both safe and effective can boost immunity without having adverse side effects. They can aid in lowering the required dose of antigen and enhancing the vaccine's overall effectiveness. Adjuvants must, however, undergo thorough testing to ensure their efficacy and safety [204]. There are noted challenges during vaccine development due to increasing production levels without sacrificing the vaccine's uniformity and quality. Transferring laboratory-scale production procedures to industrial-scale manufacturing is necessary to maintain the vaccine's safety and effectiveness even when produced in significant quantities. Maintaining uniformity among batches is crucial [205].

### Transportation and storage

There are noted challenges in guaranteeing the vaccinations' stability while being transported and stored. Many vaccinations must be refrigerated, and others must be stored at extremely low temperatures. Maintaining these conditions along the supply chain is challenging, especially in environments with limited resources [206].In addition to offering a vaccination with high efficacy and no logistical difficulties during distribution, a study by Waded S et al. found that a significant logistical mode of distribution is required. This is due to the ease of infection and mutation of SARS-CoV-2 [207]. This resembles the influenza virus, which is susceptible to mutation and necessitates a yearly update of vaccine strains [208].

By guidelines published by the World Health Organization (WHO), vaccine manufacturer, CDC (Centers for Disease Control and Prevention), and FDA, a study by Nugroho et al. compiled several distribution, storage, and packaging requirements. In addition, this study is anticipated to help technology developers (engineers), expedition/logistics businesspeople, legislators, authors, and field implementers carry out the immunization campaign. It is crucial because stakeholders need to be aware of vaccination management [209].

Antibodies generated by vaccinations can become resistant to pathogens through evolution. Certain infections with high mutation rates have the potential to produce strains that are resistant to vaccinations [210]. To solve this issue, vaccines must be updated and monitored continuously. Furthermore, a study by Dan et al. noted there is a challenge in the production of the HIV vaccine since HIV-1-positive individuals are unable to eradicate the virus; another major obstacle is the absence of distinct immunological correlates of protection in humans [211].

### Overcoming immunosuppression

Overcoming immunosuppression in the context of vaccine development presents significant challenges, especially in populations with either compromised immune systems like cancer patients or transplant recipients or autoimmunity [212]. The mechanism by which immunosuppression can negatively impact the effectiveness of vaccines lies in its ability to hinder the body from producing a proper immune response, compromising our approach to being well-protected [213]. Immune-compromised hosts are likely to have attenuated or insufficient immune responses; hence, prophylactic (therapeutic) vaccines may not have as much effect [214], leading to partial protection against infection or insufficient therapeutic benefit in diseases like cancer [215]. Developing vaccines for immunosuppressed patients [216] brings a problematic trade-off between benefit and harm [217]. These include potential antibody-dependent enhancement problems with vaccines that use live attenuated viruses or other potent immunostimulatory adjuvants, and such individuals may have an exaggerated immune response from a vaccine [218]. Immunosuppression may be affected by immunosuppressant type, and the degree of severity can vary greatly (e.g., medication-induced, disease-mediated). Such variation makes designing vaccines that will work for all individuals in these groups more challenging [219] strategies for overcoming immunosuppression are as follows.

#### Optimized vaccine formulations

One approach is to develop vaccines with enhanced immunogenicity. Some include designing vaccines that provoke more robust immune responses, such as modern Adjuvants, even in immunocompromised persons. Higher antigens or booster doses may also be necessary for an immunity percentage [220].

#### Targeted vaccine delivery

Innovative delivery systems, such as nanoparticle-based vaccines or vaccines delivered directly to immunocompromised cells, will improve the outcome in immunosuppressed persons [221] by targeting the areas of weakness that need immunological surveillance and protection [134].

#### Personalized vaccination schedules

It is possible to alter the vaccination schedule depending on the specific level of immunosuppression by either increasing the intervals between the vaccines or diversifying the type of vaccine given to an individual [222]. In the case of therapeutic vaccines, an example of which is cancer immunotherapy [223], using adjuvants such as Immunomodulatory agents [224] that mitigate the immunosuppressive pathways tends to boost the vaccine's effectiveness [225]. Monitoring immune responses after vaccine administration in the immunosuppressed population helps to make booster doses or other measures to maintain the protection level [226].

### Vaccine hesitancy

Vaccine hesitancy is a multi-faceted issue where safety is just one among several concerns that people may have. Others are cultural and traditional practices, fear of side effects, e.g., vaccination causes infertility, lack of health information, or misinformation. Distrust in health caregivers or other systems, cost, and geographical location [227]. Communication is a critical factor in managing these concerns and has been reported to lead to increased vaccine acceptance [228]. This hesitancy is not confined to high-income countries but is a complex, evolving global issue that varies significantly across different regions [229]. Immunization managers in the WHO regions have said public hesitancy affects some rural ethnic minorities and remote localities. However, elsewhere, there has been mistrust among wealthy city residents over vaccine safety [230]. There is also concern regarding religious or philosophical beliefs [231]. The factors that help or hinder the acceptance of vaccines are, for example, education is a factor. In contrast, in one setting, its effects may have a negative influence; in other settings, its effects may be optimistic. An example of how such beliefs can affect an individual is the fear of needles, leading to rejection of vaccination [232].

## Ethical and regulatory considerations in vaccine development

During vaccine development, distribution, and implementation of vaccinations, the four bioethics principles should be considered: justice, non-maleficence, autonomy, and beneficence. There are usually some challenges during the implementation of the ethics [233]. Distributive justice, rights-based justice, and legal justice are the three components of a fair decision that make up justice. Distributive justice refers to the equitable allocation of limited resources to all; equality denotes the lack of avoidable or compensable differences between various groups, nations, racial and ethnic groups, and societies at large [234].

Concerning the justice principle, the limited supply of COVID-19 vaccines that have been licensed, the strong demand from many nations, and nationalism, it may be extremely difficult or even impossible to distribute vaccines equitably [235]. According to the Adejumo et al. study, concerns like the differences in the economic standing of the nation's signing contracts with vaccine manufacturers and the lack of logistical support should not stand in the way of guaranteeing universal access to vaccines in line with the sustainable development goals—concerns like the elderly and healthcare workers being prioritized [236].

Vaccine makers must consider inclusion and exclusion criteria, such as laboratory and clinical evaluations when conducting clinical trials to include healthy people in the study [237]. As soon as the sponsors and health department inspectors verify safety and temporary efficacy, participants receiving a placebo in a clinical trial should have the right to obtain the vaccine, which should be guaranteed and safeguarded. This right should also be mentioned in the informed consent forms [238].

The existence of a comprehensive treatment facility is another problem, as it guarantees trial participants access to care and treatment in the event of any significant adverse events connected to the results of clinical trials [239]. The right to autonomy in healthcare refers to the patient's ability to make decisions about their treatment plans based on their values, attitudes, and assessments [240]. Furthermore, To "not harm" the essential and foundational tenets of medical care and research is to adhere to non-maleficence, sometimes known as "not to harm" [241].The Advisory Committee states that while allocating the COVID-19 vaccine, consideration should be given to four principles: maximizing benefits and reducing harm, promoting fairness, alleviating health inequities, and encouraging transparency [242].

The principles of beneficence and non-maleficence must be approached holistically by the notion of beneficence. Public gains thus outweigh public harms. Thus, the beneficence concept merits special consideration in clinical trials and immunization programs. In order to do this, researchers should ensure that the cumulative benefits of public health interventions surpass the drawbacks and play a significant role in containing and preventing the COVID-19 outbreak, both during the vaccine development and immunization stages [243].

Furthermore, the concept of beneficence may impact the principle of autonomy in the event of a national pandemic threat. In order to vaccinate children and adolescents, accurate information regarding the immunogenicity of vaccines should be available [233]. The public's willingness to accept the vaccine may be impacted by a lack of faith in the vaccine's efficacy and safety resulting from mistrust of scientific research, a lack of proof and information, exposure to false information about the vaccine, fear of politicization, and pharmaceutical industry misuse of the vaccine [244]. Consequently, a reliable information system must be in place for the community to make an informed decision regarding vaccinations [245].

## Conclusion and future prospects

Finding vaccination platforms capable of achieving high immunogenicity is essential, even if therapeutic cancer vaccines represent an intriguing new frontier to overcome the long-standing cancer treatment conundrum [246]. Furthermore, individual differences in tumor antigens must be addressed for an improved anti-tumor response. In order to improve clinical results, future research should concentrate on refining vaccination platforms and optimizing combination therapy to increase immunogenicity [247]. Delivering cargo with predetermined release kinetics and duration of impact to a target location or site is the goal of a drug/vaccine delivery system. As vaccination research becomes more specialized and focused, we must constantly create new delivery methods to keep up with the times. While targeting and efficiency in delivery systems are essential, the safety and practicality of clinical translation of the delivery system itself should receive greater emphasis [248].

Prospective progress toward the clinical effectiveness of therapeutic cancer vaccines is focused on multiple critical domains, such as detecting immunogenic neoantigens, refining vectors and delivery systems, and overcoming the immunosuppressive tumor microenvironment. The goal of ongoing research is to improve vaccine technology. This includes investigating various expression forms, enhancing co-stimulation elements, and determining appropriate prime-boost strategies [247]. Developing a therapeutic or preventive HIV vaccine is still critically important and will require ongoing research from all scientists [201]. Their widespread use in the HIV vaccine was constrained by safety concerns and their incapacity to produce bNAbs. Consequently, scientists are now focusing more on a few cutting-edge vaccination approaches. The consensus or mosaic immunogens were created by in silico analysis of global HIV-1 sequences to give maximum coverage of viral sequence variety and address the genetic diversity of HIV-1 strains circulating worldwide [202].

In order to prevent unintended reactions, peptide-based vaccinations may also enable the choice of adjuvants that effectively modulate the immune system as needed. Depending on further research, this might be used as a better option than the existing mRNA, viral vector, and dormant entire virus vaccines [249]. Minimum vaccine-associated effects will be crucial as risk–benefit ratios become more contentious as disease prevalence drops. However, achieving zero risk is unachievable, and there will always be a conflict between the imperatives of public health and the regulatory need to mitigate even hypothetical and distant threats [250].

Motivated by their success in COVID-19, BioNtech is developing a vaccine based on mRNA technology and the R21 and PfSPZ vaccines. This strategy might solve the difficulties in developing a malaria vaccine, such as the parasite's ability to evade immune defenses. An mRNA malaria vaccine is anticipated to be very effective, simple to produce, and safe for everyone [251]. Patients with prevalent infections may also be predicted to be free of infections caused by other forms of HPV. Prophylactic HPV vaccinations to prevent disease caused by residual disease or neoplastic cells that persist after therapy have limited scientific validity. Additional research, as well as clinical trials, are required [252]. The future of biomedical science in vaccine development holds promise, with anticipated advancements in personalized medicine, nanotechnology, and artificial intelligence (AI) poised to revolutionize research and clinical practice [253].

The transformative impact of biomedical science on vaccine development is evident through advances in both prophylactic and therapeutic approaches. With significant progress in virology, immunology, and molecular biology, innovations like mRNA vaccines and reverse vaccinology have reshaped how vaccines are designed, making them more effective and allowing rapid responses to emerging health threats. These developments have paved the way for a new era of precision in vaccine creation. Despite these advancements, the journey remains complex. Challenges such as stringent regulatory frameworks, emerging diseases, market uncertainties, and vaccine hesitancy still pose significant hurdles. Addressing these requires a concerted effort from scientists, healthcare professionals, regulators, and global public health organizations. Public education and trust-building initiatives will play a critical role in ensuring broader acceptance and uptake of vaccines. The future of vaccine development looks promising with the integration of cutting-edge technologies like AI, nanotechnology, and personalized medicine. These advancements can revolutionize vaccine production by shortening development timelines, tailoring vaccines to individual needs, and improving overall clinical outcomes. As the field progresses, interdisciplinary collaboration will remain essential to overcoming existing barriers and fostering innovation. By leveraging ongoing scientific breakthroughs, the potential for more efficient, accessible, and effective vaccines becomes increasingly tangible. These innovations promise to improve global health and ensure a more resilient and prepared response to future pandemics and infectious disease threats. The continuous evolution of the biomedical field will be vital in shaping a healthier future for all.

## Data Availability

Not applicable.

## Abbreviations

RNA: Ribonucleic Acid
mRNA: Messenger Ribonucleic Acid
DNA: Deoxyribonucleic Acid
HIV: Human Immunodeficiency Virus
HPV: Human Papillomavirus
IND: Investigational New Drug
FDA: Food and Drug Administration
ABM: Agent-based Modelling
NITAG: National Immunization Technical Advisory Group
DCs: Dendritic Cells
TB: Tuberculosis
GSK: GlaxoSmithKline
BCG: Bacillus Calmette-Guerin
ART: Antiretroviral Therapy
CIM: Center of Molecular Immunology
EGF: Epidermal Growth Factor
PRRs: Pattern Recognition Receptors
APCs: Antigen-Presenting cells
LNPs: Lipid Nanoparticles
HA: Hemagglutinin
CMV: Cytomegalovirus
RSV: Respiratory Syncytial Virus
IRVs: Inactivated Rabies Vaccines
TAAS: Tumor-Associated Antigens
GBM: Glioblastoma
NSCLCs: Non-small Cell Lung Cancers
TNBC: Triple Negative Breast Cancer
CTL: Cytotoxic T cell
CFTR: Cystic Fibrosis Transmembrane Conductance Regulator
NTPs: Nucleotide Triphosphates
VZV: Varicella Zoster Virus
VLPs: Virus-like Particles
HBsAg: Hepatitis B surface Antigen
DSB: Double-Strand Break
AAV: Adeno-associated Virus
RSVF: Respiratory Syncytial Virus Fusion
RBD: Receptor-Binding Domain
CDC: Centers for Disease Control and Prevention
WHO: World Health Organization
AI: Artificial Intelligence
